# Probe‐Based Mechanical Data Storage on Polymers Made by Inverse Vulcanization

**DOI:** 10.1002/advs.202409438

**Published:** 2024-12-16

**Authors:** Abigail K. Mann, Samuel J. Tonkin, Pankaj Sharma, Christopher T. Gibson, Justin M. Chalker

**Affiliations:** ^1^ Institute for Nanoscale Science and Technology College of Science and Engineering Flinders University Bedford Park South Australia 5042 Australia; ^2^ College of Science and Engineering Flinders University Bedford Park South Australia 5042 Australia; ^3^ ARC Centre of Excellence in Future Low Energy Electronics Technologies (FLEET) UNSW Sydney Sydney NSW 2052 Australia; ^4^ Flinders Microscopy and Microanalysis College of Science and Engineering Flinders University Bedford Park Adelaide South Australia 5042 Australia; ^5^ Adelaide Microscopy The University of Adelaide Adelaide South Australia 5000 Australia

**Keywords:** atomic force microscopy, inverse vulcanization, polysulfide, probe‐based data storage, sulfur polymer

## Abstract

Big data and artificial intelligence are driving increasing demand for high‐density data storage. Probe‐based data storage, such as mechanical storage using an atomic force microscope tip, is a potential solution with storage densities exceeding hard disks. However, the storage medium must be modifiable on the nanoscale. While polymers are promising storage media, they face challenges with synthesis, erasing temperatures, and stability. Here, a low‐cost and robust polymer system is reported that allows repeated writing, reading and erasing. The polymer is made by inverse vulcanization, providing a network of S─S bonds that can be broken and re‐formed repeatedly. This property is leveraged in mechanical indentation to encode information, and thermal S─S metathesis and polymer re‐flow to erase. Exquisite control of indentation depth is possible over 1–30 nm. This control enables data encoding not just as a function of the presence or absence of an indent, but also indentation depth. This ternary coding increases the data density four‐fold over binary coding. Furthermore, the coding can be done at room temperature which is rare for mechanical information storage. The low cost, ease of synthesis, and dynamic S─S bonds in these polymers are a promising advance in polymer storage media for probe‐based data storage.

## Graphical abstract

**Write, Read, Erase, Repeat**. A polymer made from low‐cost sulfur and dicyclopentadiene is used as a novel data storage medium for probe‐based computing systems. The information is encoded mechanically at room temperature, with nanoscale precision, and can be erased in seconds with heating. Ternary coding is demonstrated, which increases the storage density fourfold compared to binary coding, exceeding storage densities of hard disks.

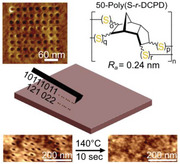

## 1 Introduction

The proliferation of digital information has led to expanded data storage requirements, with annual global data generation on the zettabyte scale.^[^ ^1^, ^2^ ^]^ Hard disk drives,^[^ ^3^ ^]^ optical data storage,^[^ ^4^ ^]^ solid‐state drives, and flash memory^[^ ^5^ ^]^ are widely used technologies, but all have limitations in data storage density. The superparamagnetic limit constrains the size reduction of the magnetic grain in hard disks,^[^ ^6^ ^]^ diffraction limits restrict the storage capacity in optical discs,^[^ ^4^, ^7^ ^]^ and the reduction of the size of flash cells and solid‐state drives is complicated by physical scaling limits.^[^ ^8^ ^]^

Probe‐based memory is one potential strategy for addressing the need for achieving high‐density non‐volatile data storage.^[^ ^9^ ^]^ In this approach, a nanoscale probe is used to record and read data on the storage material. The probe comprises a fine tip mounted on a cantilever of an atomic force microscope (AFM) allowing recording and visualization of the material's morphology, and magnetic, polar, and electronic states with nanoscale precision.^[^ ^10^ ^]^ The encoded data in probe‐based information storage can take the form of nanoscale modifications of the storage medium. The nanoscale modifications act as single information cells, such as pits (mechanical data storage), crystalline and amorphous domains (phase‐change storage), or areas of precise magnetic or electrical polarization (magnetic and ferroelectric storage, respectively).^[^ ^9^, ^10^ ^]^

For mechanical information storage, polymers have attracted significant interest as storage media due to their low cost, ease of fabrication, and chemical and physical versatility.^[^ ^11^ ^]^ To encode data, the scanning probe is used to modify or create a pit or indent on the polymer surface, where an indent can represent a cell state of “1” and its absence denotes a cell state of “0” in binary coding. Creating indents on the polymer surface typically requires a heated probe to provoke the required thermomechanical^[^ ^12^, ^13^, ^14^ ^]^ or chemical^[^ ^15^ ^]^ modification. Baroplastic polymers have also been reported with surfaces that can be modified without heating through highly localized nanomechanical pressures exerted by a probe tip, triggering a phase transition between a crystalline and disordered state.^[^ ^16^ ^]^ Because of the high spatial resolution of scanning probes, data densities of 4 Tb/in^2^ have been encoded on polymer surfaces, exceeding typical areal recording densities of hard disk drives.^[^ ^17^ ^]^ This high data density has prompted interest in mechanical data storage for archival applications.^[^ ^18^ ^]^ To address the issue of slow reading and writing in these scanning probe systems, arrays of cantilevers can be used for rapid and parallel processing.^[^ ^13^, ^14^, ^19^ ^]^ Alternatively, massively parallel atomic force microscopy using optical detection methods have recently been reported that dramatically enhance the rate and scale of scanning probe imaging.^[^ ^20^ ^]^ Erasing the nanoindentations can be achieved by heating the polymer above its glass transition temperature.^[^ ^12^, ^16^ ^]^

To realize the commercial potential of polymers as data storage media, these materials need to be low‐cost and easy to make and process. The polymer must also have thermomechanical or chemical properties that allow for high precision, nanoscale modification with the probe. If data encoding can be done without heating, such a system would simplify the data encoding protocols, and benefit from lower energy consumption. Additionally, the polymer surface modifications need to be persistent and stable for high‐fidelity storage, but also easily erasable. The polymer must also be robust enough to undergo many cycles of reading, writing, and erasing.

In this study, we report the first demonstration of polysulfide polymers as storage media for probe‐based data systems. Mechanical information storage was achieved using probe‐induced nanomechanical force which caused controlled nanoscale morphology changes of the polysulfide polymers. This class of polymers can be made by the copolymerization of sulfur and organic dienes, a process referred to as *inverse vulcanization*.^[^ ^21^ ^]^ While mechanistically complex,^[^ ^22^ ^]^ this technique is operationally simple and inexpensive in providing polymers with networks of S─S bonds.^[^ ^23^, ^24^, ^25^, ^26^, ^27^ ^]^ These sulfur‐rich polymers have been explored in a number of creative applications such as energy storage,^[^ ^21^, ^28^, ^29^, ^30^ ^]^ infrared optics,^[^ ^31^, ^32^, ^33^, ^34^, ^35^ ^]^ precious metal recovery,^[^ ^36^ ^]^ and environmental remediation.^[^ ^37^, ^38^, ^39^, ^40^, ^41^, ^42^ ^]^ The S─S bonds in polymers made by inverse vulcanization or related processes can also be broken and reformed when exposed to thermal,^[^ ^43^, ^44^, ^45^ ^]^ thermomechanical,^[^ ^46^, ^47^ ^]^ chemical,^[^ ^48^, ^49^ ^]^ or photochemical^[^ ^36^, ^50^ ^]^ stimuli. This dynamic covalent character has been exploited in polymer repair and recycling,^[^ ^43^, ^48^, ^49^ ^]^ composite fabrication,^[^ ^44^, ^51^ ^]^ chemically activated adhesives,^[^ ^48^, ^52^ ^]^ depolymerization and monomer recycling,^[^ ^53^ ^]^ and lithography.^[^ ^50^ ^]^ The dynamic nature of the S─S bond in these polymers, along with the ease of synthesis and low cost, prompted us to evaluate these materials as data storage media. We hypothesized that the topological structure of the polymer surface would be amenable to mechanical information storage using an AFM cantilever and that these modifications could be erased by thermal reorganization of the dynamic polysulfide network. In validating this hypothesis, inverse vulcanization can now be considered a path to data storage polymers.

## 2 Results and Discussion

**Figure 1:**
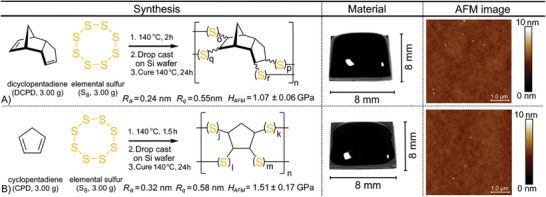
A) Synthesis of 50‐poly(S*‐r*‐DCPD) and B) 50‐poly(S‐*r*‐CPD). Both materials could be drop cast and cured to form a hard, smooth surface.

Two polymers were prepared by reacting elemental sulfur with either dicylopentadiene (DCPD)^[^ ^38^, ^54^ ^]^ or cyclopentadiene (CPD) (**Figure** **1**; Figures S5–S7, Supporting Information).^[^ ^34^ ^]^ These polymers are referred to as 50‐poly(S‐*r*‐DCPD) and 50‐poly(S‐*r*‐CPD), where 50 designates the mass percentage of sulfur, *r* indicates the process is a random copolymerization, and DCPD and CPD refer to the organic monomer. These polymers were selected because they contain the requisite S─S bonds for dynamic covalent properties, and both are low‐cost, easy to prepare, and can be drop cast and cured to hard, smooth surfaces. Multigram syntheses of both polymers have been reported.^[^ ^34^, ^54^ ^]^

For both polymers, AFM was used to quantify surface roughness (Figure S8, Supporting Information), with *R_a_* = 0.24 ± 0.03 nm and *R_q_* = 0.55 ± 0.21 nm for 50‐poly(S‐*r*‐DCPD) and *R_a_* = 0.32 ± 0.14 nm and *R_q_* = 0.58 ± 0.39 nm for 50‐poly(S‐*r*‐CPD), all measured over a 5 × 5 µm area. This low surface roughness is critical for mechanical information storage using nanoscale indentations. The Young's modulus and surface hardness were also quantified (Figures S9 and S10, Supporting Information), as these properties will influence the tip lifetime of the probe.^[^ ^55^ ^]^ For 50‐poly(S‐*r*‐DCPD), these values were 1.78 ± 0.34, GPa and 1.07 ± 0.06 GPa, respectively. For 50‐poly(S‐*r*‐CPD) the Young's modulus was 1.09 ± 0.27 GPa and the surface hardness was 1.51 ± 0.17 GPa.

**Figure 2:**
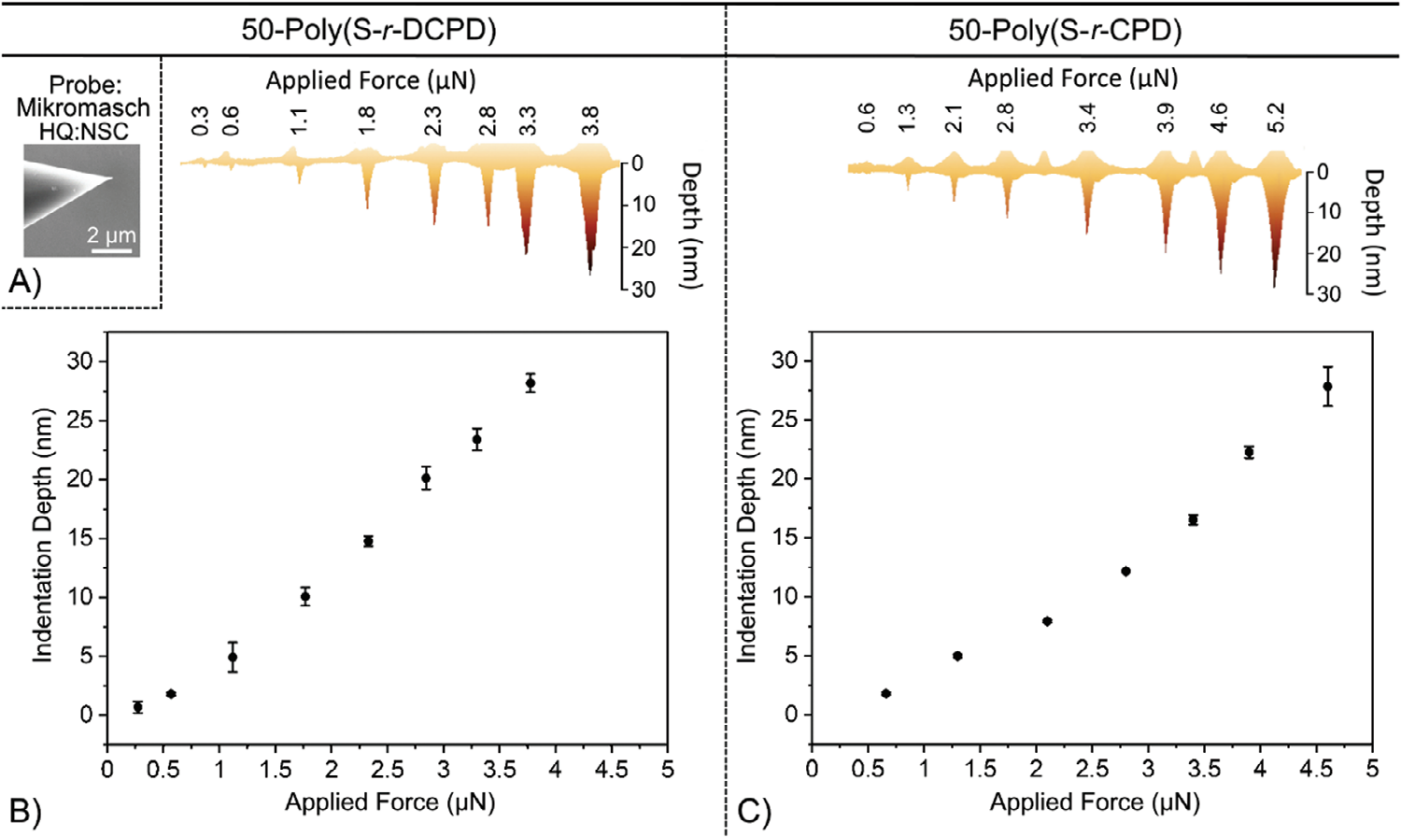
The applied force of the Mikromasch HQ:NSC AFM tip (A) was correlated with the resulting indentation depth for both 50‐poly(S‐*r*‐DCPD) (B) and 50‐poly(S‐*r*‐CPD) (C). Precise control of the indentation depth was observed for depths ranging from 1 to 30 nm. The image of the AFM tip was acquired after using to make the nanoindentations and no evidence of wear or polymer contamination was observed for this series of experiments (Figure S13, Supporting Information).

With topographically smooth polymer surfaces in hand, nanoindentation was tested next with the goal of understanding how the depth of the indentation varied with the applied force of the AFM tip. Accordingly, a range of forces (0.6–3.8 µN) were applied using a Mikromasch (HQ:NSC 15) AFM tip across the surface of each polymer. The resulting nanoindentations for both polymers were then imaged by AFM (**Figure** **2**). The depth of the indentation was correlated with the applied force, with a high level of control for indentations ranging from <1 to 30 nm (Figures S11 and S12, Supporting Information).

**Figure 3:**
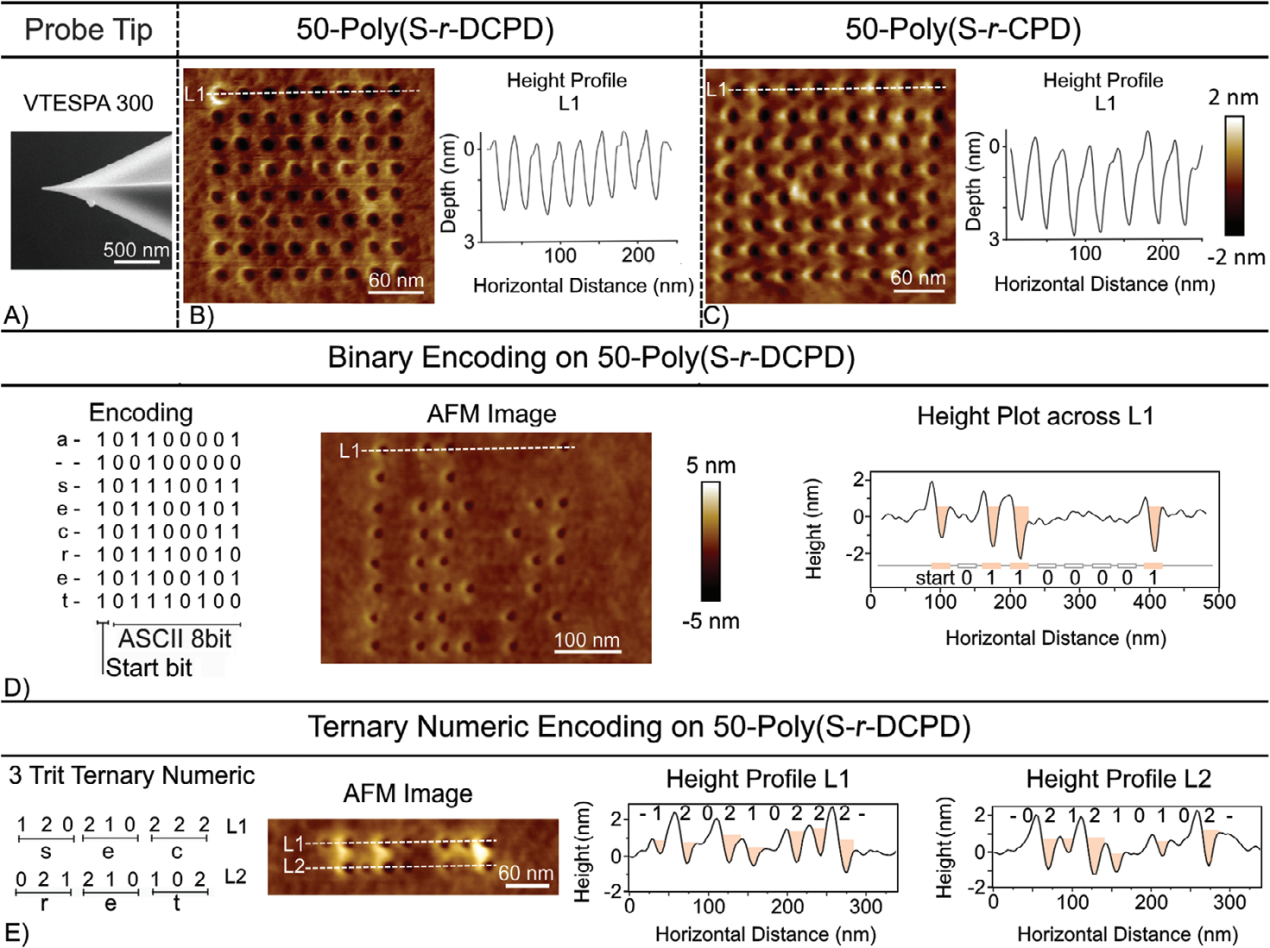
A) VTESPA 300 probe tip used to make precision indentations. B) AFM image of the nanopatterned surface of 50‐poly(S‐*r*‐DCPD) with a calculated data density of 0.9 Tb/in^2^. C) AFM image of the nanopatterned surface of 50‐poly(S‐*r*‐CPD) with a calculated data density of 0.9 Tb/in^2^. D) Mechanical storage of a phrase (“a secret”) encoded in binary on 50‐poly(S‐*r*‐DCPD) (1 character: 7050 nm^2^) E) Mechanical storage of a word (secret) encoded with a ternary code on 50‐poly(S‐*r*‐DCPD) (1 character: 1627 nm^2^).

Encouraged by the control over the nanoindentation depth, the process was next modified to increase storage density. First, an AFM tip with a higher aspect ratio (Bruker VTESPA 300) was used next so that more indents could be made per unit area (**Figure** **3**A; Figure S14, Supporting Information). Less force (≈200 nN) was required to generate 3 nm indentations with this tip. Again, exquisite control of the indentation depth was observed. Arrays of indents in both polymers were generated, with the widest part of the indent measuring 15 ± 1 nm and the distance between the center of the indents set to 25 ± 2 nm (the pitch). For a binary system, these arrays equate to an unoptimized data density of ≈0.9 Tb/in^2^ for both polymers (Figure 3B,C). A theoretical density of 1.5 Tb/in^2^ is possible in this system by further reducing the pitch to 22 nm, which is the minimum based on the indentation size and polymer buildup on the edge of the indentations.

Next, the precise control of the depth of the indent was leveraged to create a simplified version of a ternary numeric coding system. This was demonstrated where cells could have 3 states: “0” represented an area of no indentation, “1” was assigned to indentations ranging from 0.3 to 1.0 nm in depth, and “2” was assigned to indentations of 1.5–2.5 nm (Figures S15 and S16, Supporting Information). This strategy further increased the data storage density over the surface of the polymer, as shown by comparison to a binary counterpart (Figure 3D,E). In these comparisons, 50‐poly(S‐*r*‐DCPD) was used as the polymer substrate. In Figure 3D, the word “secret” was encoded using ASCII 8 bit, with 2 nm indents encoded in an array, where each line of dots represents a letter in the word. A start bit was also provided as an indent for each line in this binary system. In the ternary coding (Figure 3E), each letter (set of three cells) was encoded over a smaller area (≈25% the area required for binary coding), as the depth of the indent also includes information and allows a letter to be represented by only three indentations. This data storage method therefore corresponds to a four‐fold increase in data storage density in comparison to the binary coding method across the same surface. The binary storage allows each cell to have 2 states, and 8 cells can represent 256 characters. The ternary encoding method allows each cell to have 3 states and can represent 6561 characters across 8 cells, increasing the character limit 25‐fold. Additional examples of encoding using a ternary system for three different indentation depths are provided in the Supporting Information (Figures S17 and S18, Supporting Information). Importantly, the indentations were stable and did not change, as measured by AFM imaging, over a period of three months (Figures S19 and S20, Supporting Information). This demonstration of ternary coding reflects the precision and reproducibility in the installation and reading of the indents with nanoscale resolution. This ability is a rare example of such multi‐level recording strategies in mechanical data storage.^[^ ^18^ ^]^

**Figure 4:**
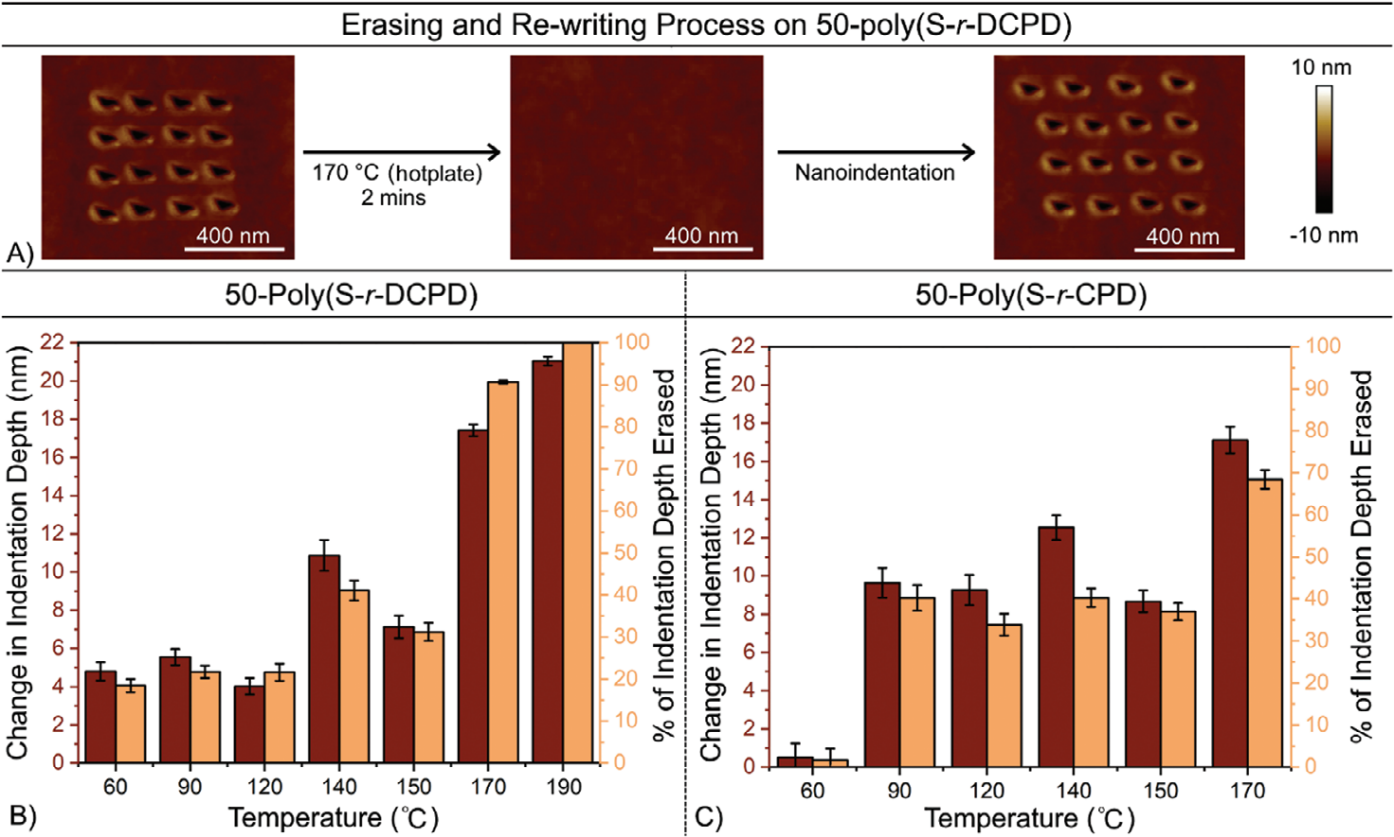
A) Erasing and re‐writing demonstration on 50‐poly(S‐*r*‐DCPD). B) Erasing indentations in 50‐poly(S‐*r*‐DCPD) required heating to at least 170 °C. C) Heating also erased indents in 50‐poly(S‐*r*‐CPD), but the erasing process was not complete for this polymer even at 170 °C. All indentations in Figure 4 were ≈18–27 nm deep and installed with the Mikromasch HQ:NSC tip.

With high‐density writing and reading methods established for the polymer systems, data erasing was investigated next (Figures S21–S24, Supporting Information). It was hypothesized that the indentations could be erased by simply heating the polymer and initiating S─S metathesis that allows the polymer to reflow and fill in the indentations. First, 4 × 4 arrays of indentations with depths of 18–27 nm were patterned on the surface of seven samples of 50‐Poly(S*‐r‐*DCPD) and 50‐Poly(S*‐r‐*CPD) drop cast and cured on silicon substrate. AFM images were acquired before and after heating to examine change in depth of indentations. For both polymer samples, heating to 140 °C on a hotplate resulted in a significant change in the indentation depth, and heating to 170 °C for 2 min could reliably and completely erase the indentations in the 50‐Poly(S*‐r‐*DCPD) sample. Importantly, the area that was erased could be re‐used to encode new data (**Figure** **4**; Figures S25 and S26, Supporting Information).

The 50‐poly(S‐*r*‐DCPD) was more robust in the writing, erasing, and re‐writing process, than 50‐poly(S‐*r*‐CPD), perhaps because of the former's higher *T*_g_ (>100 °C) in comparison to the *T*_g_ of the latter (40 °C). The higher *T*_g_ of 50‐poly(S‐*r*‐DCPD) may be better suited for erasing with the onset of this process starting at 140 °C or higher, while maintaining a persistent morphology at lower temperatures. 50‐Poly(S‐*r*‐DCPD) was therefore used in subsequent experiments in encoding data on polymer films. Thin polymer films were studied next as this architecture is typically required for probe‐based storage devices.^[^ ^9^, ^10^ ^]^ Furthermore, for such devices, it would need to be established what forces are required for nanoindentation on a film of 50‐poly(S‐*r*‐DCPD) and the temperature and time required for erasing data, which was anticipated to be lower in thin polymer films.

**Figure 5:**
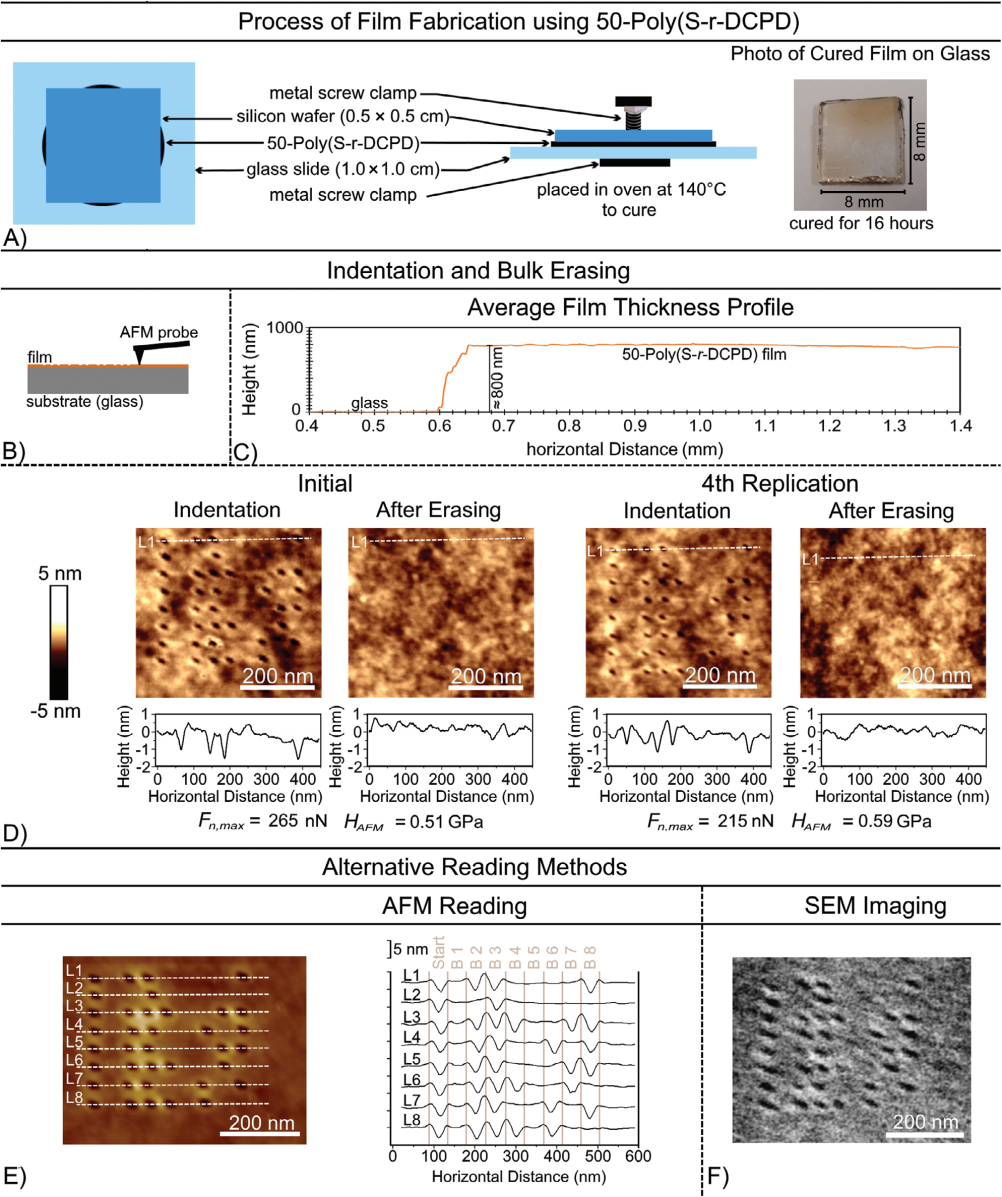
A) Process for casting and curing a film of 50‐poly(S‐*r*‐DCPD). The cured film (≈800 nm thick) is transparent on the glass substrate, which contrasts to the cured bulk polymer, which is black. B) Schematic for making nanoindentations in the polymer film. C) Stylus profilometry measurements indicating a film thickness of 800 nm on the glass substrate. D) Encoding and erasing “a secret” on the polymer film. The erasing was complete after heating for 10 s at 140 °C. The writing and erasing were repeated four times (Bruker VTESPA 300 tip). E) Writing and reading the code using AFM on the 50‐poly(S‐*r*‐DCPD) film (Bruker VTESPA 300 tip). The vertical axis displays height profile along L1‐8 with the distance between each tick mark representing a depth of 5 nm. F) Imaging the nanoindentations by scanning electron microscopy (SEM) constitutes an orthogonal method of surface characterization, and a rapid method of reading encoding information.

Accordingly, a film of 50‐poly(S*‐r‐*DCPD) was made by sandwiching one drop of the liquid pre‐polymer (≈10 mg) between a silicon wafer and precut glass slide with a screw clamp (**Figure** **5**A–C; Figures S27–S29, Supporting Information). After curing for 12 h, the silicon wafer was removed, and the sample was then heated to 150 °C for 2 min to thermally “polish” the film surface. This technique provided films ≈800 nm in thickness (measured by stylus profilometry, Figure 5C) with surface roughness of *R_a_* = 0.30 nm and *R_q_ =* 0.43 nm. The surface hardness of the film was 0.51 GPa, as determined by AFM nanoindentation, which was less than the hardness of the bulk polymer surface which was on the order of 1 GPa. This method of making polymer films was more effective than spin coating in accessing uniform films of cured 50‐poly(S‐*r*‐DCPD) (Figure S37, Supporting Information).

Gratifyingly, the polymer film could be modified using the VTESPA 300 tip with a force of 215–265 nN to encode information as indents ≈2 nm deep. Notably, the force required to make the indents was approximately the same for both the polymer film and bulk 50‐poly(S‐*r*‐DCPD). The polymer build‐up around the indentations was also much smaller in the film (2‐4 nm extended radius, 0.2 nm height). Moreover, these indents could be erased by heating to 140 °C for a mere 10 s. Four iterations of writing, reading, erasing and re‐writing were demonstrated using this method (Figure 5D). Similar outcomes were also observed for films that were 550 nm or 7.5 µm thick. Additional studies on the polymer film stability were also carried out by subjecting samples of the 50‐poly(S‐*r*‐DCPD) to 30 rounds of heating (140 °C, 10 s) and cooling to room temperature. No change was observed in terms of surface roughness and elemental composition, and no elemental sulfur was formed in this cycling process (Figures S33 and S34, Supporting Information).

Finally, the code in the polymer film was also imaged by scanning electron microscopy as an alternative readout method (Figure 5E,F; Figures S30–S32, Supporting Information). While depth information cannot be accurately measured in the SEM imaging, the nanoindentations could clearly be resolved for decoding binary data, providing a reading method more rapid (≈0.6 kb s^−1^) and orthogonal to probe‐based decoding.

## 3 Conclusion

Polymers made by inverse vulcanization were used, for the first time, in mechanical data storage. These polymers contain polysulfide networks with S─S bonds that can be broken and re‐formed, which facilitates writing, reading, and erasing by the probe‐based system. The control of the depth of the indentations allowed for ternary coding, which increased storage density four‐fold in comparison to binary coding. Installing the nanoindentations at room temperature is also rare and the polymer used here is far simpler to make and process than previous reports that rely on sophisticated baroplastic block copolymers.^[^ ^16^ ^]^ The coding could also be done on polysulfide polymer films, which reduced the erasing time to seconds. This feature is notable in reducing the energy consumption for erasing compared to other data storage polymers, but we do note that such storage systems could only operate in environments below the erasing temperature. As probe‐based data storage has the potential for higher data storage densities than alternative storage methods, this technique may be revisited in future computing technologies. The polymers reported here may provide a low‐cost and effective storage media option for mechanical storage in probe‐based memory systems. In future studies, we plan to explore orthogonal methods for manipulating the data in the polymer, such as erasing with low‐power lasers rather than heat.^[^ ^50^ ^]^ We will also explore rapid methods to copy the mechanically encoded data, such as nanoimprint lithography.^[^ ^56^ ^]^

## Conflict of Interest

Two authors (S.J.T. and J.M.C.) are inventors on a patent application that includes the synthesis and applications of copolymers made from sulfur and cyclopentadiene (AU2022900289, priority date 11 Feb 2022).

## Supporting information



Supporting Information

## Data Availability

The data that support the findings of this study are available in the supplementary material of this article.
