# Supplementary material for: Probe‐Based Mechanical Data Storage on Polymers Made by Inverse Vulcanization
Source: Adv Sci (Weinh). 2024 Dec 16;12(5):2409438. doi: 10.1002/advs.202409438 (PMC11792057; doi:10.1002/advs.202409438)
Supplement: Supplementary file 1 — Supporting Information [file ADVS-12-2409438-s001.pdf]

ADVANCED SCIENCE
Open Access

Supporting Information

for *Adv. Sci.*, DOI 10.1002/advs.202409438

Probe-Based Mechanical Data Storage on Polymers Made by Inverse Vulcanization

Abigail K. Mann, Samuel J. Tonkin, Pankaj Sharma, Christopher T. Gibson* and Justin M. Chalker**

Supporting Information

Probe-Based Mechanical Data Storage on Polymers Made by Inverse Vulcanization

Abigail K. Mann,[1,2] Samuel J. Tonkin,[1,2] Pankaj Sharma,[1,2,3]* Christopher T. Gibson[2,4,5]*, Justin M. Chalker[1,2]*

1) Institute for Nanoscale Science and Technology, College of Science and Engineering, Flinders University, Bedford Park, South Australia 5042, Australia
2) College of Science and Engineering, Flinders University, Bedford Park, South Australia 5042, Australia
3) ARC Centre of Excellence in Future Low Energy Electronics Technologies (FLEET), UNSW Sydney, NSW, 2052, Australia
4) Flinders Microscopy and Microanalysis, College of Science and Engineering, Flinders University, Bedford Park, Adelaide, South Australia 5042, Australia
5) Adelaide Microscopy, The University of Adelaide, Adelaide, South Australia 5000, Australia

E-mail:

pankaj.sharma@flinders.edu.au

christopher.gibson@adelaide.edu.au

justin.chalker@flinders.edu.au

Table of Contents

General considerations S3

Polymer synthesis S5

Surface roughness measurement S8

Young's modulus measurement S9

Surface hardness measurement S10

Nanoindentation force and depth experiments S11

SEM and EDX analysis of AFM tips before and after indentation S13

Nanoindentation using AFM tip with high aspect ratio S14

Encoding information on 50-poly(S-*r*-DCPD) in binary and ternary S15

Storing information on 50-poly(S-*r*-DCPD) using an alternative ternary code S17

Stability of information encoded on 50-poly(S-*r*-DCPD) S19

Erasing studies S21

Re-writing data after erasing indentations on 50-poly(S-*r*-DCPD) S25

Reading, writing, and erasing data on films of 50-poly(S-*r*-DCPD) S27

SEM imaging of nanoindentations on 50-poly(S-*r*-DCPD) S30

Stability of 50-poly(S-*r*-DCPD) film over repeated heating and cooling cycles S33

Encoding information on 50-poly(S-*r*-DCPD) films of different thicknesses S35

Reproducibility in encoding information on 50-poly(S-*r*-DCPD) film S36

Spin coating trials using 50-poly(S-*r*-DCPD) S37

References S38

General Considerations

Materials

Dicyclopentadiene and sulfur were purchased from Sigma Aldrich and used as received. *N(Sb)*-doped SiO_2 wafers were purchased from D&X Co., Ltd. (Japan) and cut to size (5 mm × 5 mm and 8 mm × 8 mm) with a Accretec SS20 wafer dicer, ultrasonicated with 100% ethanol and dried under a stream of compressed air.

Scanning Electron Microscopy (SEM) with Energy Dispersive X-ray (EDX) Spectroscopy

Polymer samples were sputter coated with platinum metal (10 nm thickness, 25 mA deposition current, EmiTech K575X Sputter Coater). AFM tips were analysed as received; measurements were obtained post experiments to minimize risk of damage to AFM tips. SEM/EDX images of the samples were obtained using a FEI Inspect F50 SEM fitted with an EDAX energy dispersive X-ray detector.

AFM Imaging

Images were obtained using tapping mode in ambient atmosphere, with all parameters including setpoint, scan rate and feedback gains adjusted to optimize image quality. In order to minimize tapping force, amplitude set points during scanning were kept at 80 to 90% of the cantilever free amplitude. The AFM probes used for measurements are indicated in the protocols and include: 1) Mikromasch HQ: NSC15 Si probes with a nominal spring constant of 40 Nm^{-1} and a nominal tip radius of 8 nm. The cantilever sensitivity and spring constant were calibrated using procedures outlined in Sader et al.[1] The spring constant for cantilevers calibrated ranged between 21.9 to 35.0 N/m. 2) Bruker VTESPA-300 with a measured resonant frequency of 325.674 kHz and a calibrated spring constant of 39.0 ± 4.3 N/m and deflection sensitivity of 46 ± 5 nm/V. The scanner was calibrated in x, y and z directions using a silicon calibration grid (Bruker model number VGRP: 10 µm pitch, 180 nm depth PG: 1 µm pitch, 110 nm depth, Mikromasch model TGZ01: 3 µm pitch, 18 nm depth).

Roughness Measurements

12 images using AFM were collected of a 5 µm x 5 µm area, across 3 samples and subsequently analyzed using Nanoscope Analysis software version 2.0 to generate roughness parameters R_a and R_q.

Hardness and Elastic Modulus Measurements

Hardness and Elastic Modulus measurements were performed using a combination of Bruker Nanoscope Analysis version 2.0 software for analysis of Force curves and Gwyddion version 2.63 for characterizing the indentations formed.

AFM Nanoindentation

Nanoindentation was undertaken using a Multimode AFM with a Nanoscope V controller, with further assistance from the Point and Shoot feature of the software. The force spectroscopy section of the software was used to force the tip into the surface to generate indentations using TM (tapping mode) deflection curves. This applied a collection of force distance curves with a range of applied force, calculated through prior spring constant and deflection sensitivity calibration using the were calibrated using procedures outlined in Sader et al.[1]. Applied forces were measured from TM (Tapping Mode) deflection curves was from 0.3 μN to 5.4 μN. After indentation had been achieved, an image of the modified area, using AFM was acquired.

Stylus Profilometry

Stylus profilometry was carried out using a Bruker DektakXT stylus profilometer running on Vision64 (Bruker) software. This technique provides topographical images on areas greater than 200 μm × 200 μm with a z resolution up to 10 nm. The tip diameter was 2 μm. The load force of the stylus was 1 mg and the scan resolution of 0.2 μm. These experiments were performed in ambient conditions. Data was processed using Gwyddion 2.63.

Raman Spectroscopy

Raman spectra were collected using a XplorRA Horiba Scientific Confocal Raman microscope at an excitation laser wavelength of 532 nm at a measured power of 0.11 mW with a 50X objective (NA = 0.6) with a spectrometer resolution of 4.5 cm^{-1}. The silicon signal at 520.6 cm^{-1} was used for calibration. Spectra of polymer samples were recorded for the surface by averaging 60 accumulations with 1 sec acquisition time; this was done by acquiring single spectra across 10 different locations across a 100 μm area. The resulting spectra were processed by applying a correction to account for observed oscillations in the spectra due to absorptions by the edge filter used to suppress the Rayleigh scattering peak. Further a background was fitted and subtracted from the spectra to remove fluorescence from the sample.

Synthesis of 50-Poly(S-*r*-DCPD)

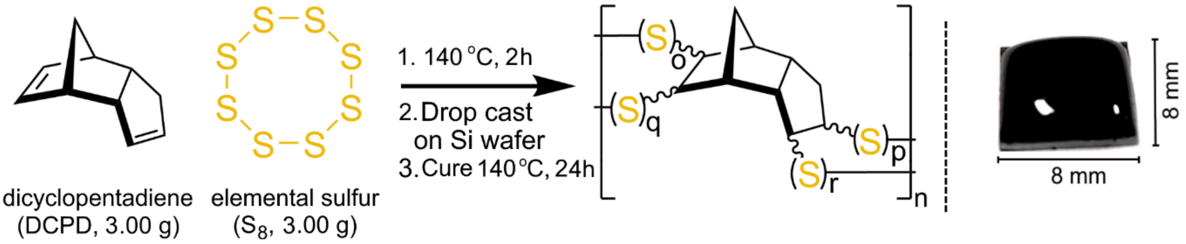

The synthesis of 50-Poly(S-*r*-DCPD) was carried out as previously described.[2] Dicyclopentadiene (DCPD) (3.00 g, 0.023 mol) was added as a solid to a pre-weighed, and flame-dried 21 mL scintillation vial equipped with a magnetic stir bar. Sulfur (3.00 g, 0.012 mol) was directly added as a powder to the vial containing DCPD. A rubber septum was used to seal the vial. The mixture was placed under an atmosphere of nitrogen and heated at 140 °C with stirring. A pre-heated magnetic stirrer/hotplate was used in combination with an aluminum vial holder and heating block to maintain a constant temperature. After 2 h, a viscous dark brown mixture was formed, which was removed from heat and exposed to air. The prepolymer (0.05 g) was directly drop cast onto a pre-cut silicon wafers before it was added to an over preheated to 140 °C. The sample was cured at 140 °C for 24 h. The cured samples were allowed to cool gradually before being removed from the oven. Samples of the polymer were analyzed using AFM to determine surface roughness.

Cyclopentadiene (CPD) synthesis

A distillation setup was prepared using a water-cooled condenser. Dicyclopentadiene (DCPD) was added to a 100 mL round bottom flask. The reaction was typically done in batches with 20 to 40 g of DCPD. The flask was placed in an oil bath and slowly heated to 180 °C with constant stirring. The conversion from DCPD to cyclopentadiene (CPD) was typically performed over 4 h. As CPD readily converts back to DCPD via a Diels Alder reaction at room temperature, the collection flask was cooled in an ice bath and the CPD was regularly removed and stored in a freezer at -3 °C. The CPD was further purified by a second distillation using a bath temperature of 70 °C. The CPD cannot be stored long term, even at reduced temperatures, as it converts back to DCPD. It was therefore important to use the CPD immediately after distillation and to regularly check its purity using ^{1}H NMR spectroscopy. The purity of the CPD was checked before any reaction was performed. The relative percentage of DCPD in the CPD precursor was calculated via the integration of the ^{1}H NMR spectra and the CPD would only be used if the DCPD content was less than 2%. Typical ^{1}H NMR spectra for pure DCPD and CPD are shown below.

Supplementary Figure S1: Stacked ^{1}H NMR spectra ($CDCl_3$, 298 K, 600 MHz) showing the synthesis of cyclopentadiene (CPD) from dicyclopentadiene (DCPD)

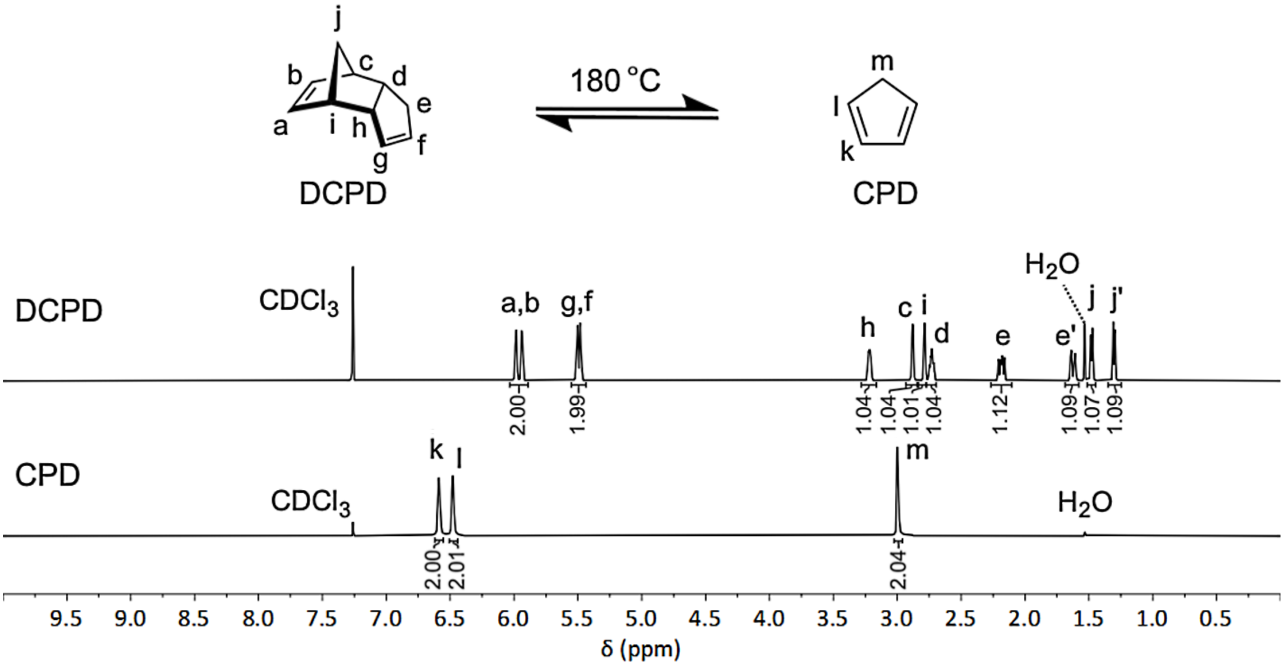

Synthesis of 50-Poly(S-*r*-CPD)

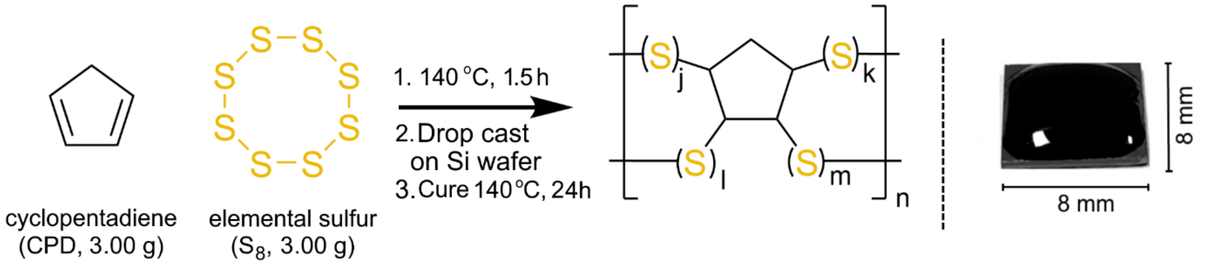

Samples of 50-poly(S-*r*-CPD) were prepared using a reaction between sulfur and cyclopentadiene (CPD) as previously described.[3] Sulfur (3.00 g, 0.012 mol) was added to a pre-weighed 21 mL scintillation vial with a magnetic stirrer. A threaded adapter was used to attach the vial to a water-cooled condenser. The sulfur was lowered into a preheated, 140 °C oil bath for 3 mins to form a yellow liquid. After this time, CPD (3.00 g, 3.71 mL, 0.045 mol) was added down the reflux condenser using a micropipette. The CPD was added slowly (150 μL additions every 3 mins) to prevent cooling crystallization of the sulfur. After the CPD addition, the reaction was stirred at 140 °C for a total of 90 minutes. Over this time, the sample slowly darkened and increased in viscosity as a low molecular weight prepolymer formed. The vial was removed from the reflux condenser and the magnetic stirrer was extracted. The prepolymer (0.05 g) was directly drop cast onto pre-cut silicon wafers before it was added to an oven to 140 °C. The samples were then cured at 140 °C for 24 h. The cured samples were allowed to cool gradually before being removed from the oven. Samples of the polymer were analyzed by AFM to determine surface roughness.

Surface roughness measurements of 50-poly(S-*r*-DCPD) and 50-poly(S-*r*-CPD)

Supplementary Figure S2: Example of AFM images of the surface of 50-poly(S-*r*-DCPD) and 50-poly(S-*r*-CPD) showing surface topology.

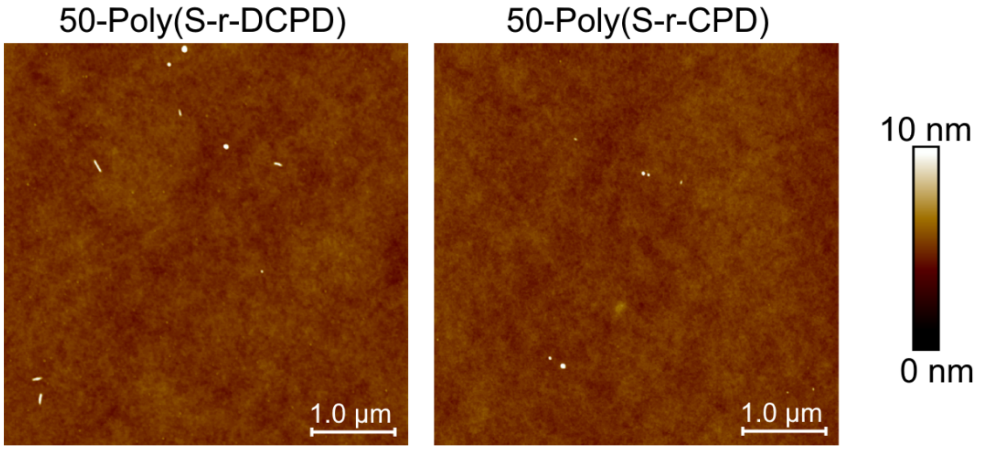

Twelve 5 μm × 5 μm area images were acquired of the polymer sample surfaces across 3 samples at locations greater than 100 μm distance between sampling sites. Roughness parameters such as average (R_a), and root mean square (RMS, R_q), were extracted through a roughness analysis performed on ach AFM image. The average of the 12 values was calculated and one standard deviation (SD) of the mean and standard error (SE) in the mean of these values is reported. Particles on the surface will tend to raise the R_q value more than the R_a value. This is demonstrated through differences between the absolute values for the R_a and R_q roughness for each AFM image the overall trends for both types of roughness analysis were similar. The roughness parameters are presented in the table below.

Supplementary Table S1: Table of average roughness parameters (R_a and R_q) of the 12 values, one standard deviation (SD) of the mean and standard error (SE) in the mean.

**Material**	R_a **(nm)**	**SD (nm)**	**SE (nm)**	R_q **(nm)**	**SD (nm)**	**SE (nm)**
**50-poly(S-*r*-DCPD)**	0.24	0.03	0.01	0.55	0.21	0.06
**50-poly(S-*r*-CPD)**	0.32	0.14	0.04	0.58	0.39	0.11

Young's modulus measurements of 50-Poly(S-*r*-DCPD) and 50-Poly(S-*r*-CPD)

AFM nanoindentation was used to determine the Young's modulus of the surface of the polymers. A total of 30 force curves were acquired at three locations across the polymer surface, each location being separated by hundreds of microns. Collected force curves were converted to indentation curves and the Hertz model was used to determine the Young's modulus. The equation for the Hertz model is given below:[4]

$$F = \frac{4}{3} E \frac{R^{1/2} \delta^{3/2}}{(1 - v^2)} \quad [1]$$

Where E = Young's modulus, F = applied force determined by the calibration of the cantilever sensitivity and spring constant[1] and extracted from the indentation curve, R = AFM tip radius of 7 nm, δ = indentation of the surface produced from the applied F, and v is the Poission ratio which is approximated as 0.3 which is a value typical for polymers.[5]

The Nanoscope software fits a curve, using the Hertz model, to the indentation curve and from this the Young's modulus is determined as a fit parameter. Only loading curves were analysed to determine the Young's modulus as outlined in Kontomaris et al.[6]

Supplementary Table S2: Young's modulus data for 50-poly(S-*r*-DCPD) and 50-poly(S-*r*-CPD):

**Material**	**Young's Modulus (GPa)**
50-poly(S-*r*-DCPD)	1.78 ± 0.34
50-poly(S-*r*-CPD)	1.09 ± 0.27

Surface hardness measurements of 50-poly(S-*r*-DCPD) and 50-poly(S-*r*-CPD)

For the surface hardness measurements, the required measurements are the maximum applied force and the projected surface area of the indentations made by the AFM tip. A total of 25 force curves were collected for these, measurements over 3 different areas of the surface, which were a minimum of 100 μm distance apart. The equation to determine surface hardness is described in the following equation:[4]

$$H_{AFM} = \frac{F_{n,\ max}}{A_p} \quad [2]$$

Where $F_{n,max}$ is the maximum applied force of the tip to the surface during indentation and A_p is the projected surface area of resulting imprint calculated using Gwyddion.

Supplementary Table S3: Surface Hardness of 50-poly(S-*r*-DCPD) and 50-poly(S-*r*-CPD):

Material	$F_{n,max}$ (μN)	Avg. A_p (nm^2)	Avg. H_{AFM} (GPa)
50-poly(S-*r*-DCPD)	2.54 ± 0.01	2388 ± 106	1.07 ± 0.06
50-poly(S-*r*-CPD)	2.57± 0.02	1719 ± 185	1.51 ± 0.17

Nanoindentation for data storage

General Method

Samples were used as prepared in the synthesis method. The following experiments were undertaken using 50-poly(S-*r*-DCPD) and 50-poly(S-*r*-CPD). Atomic force microscopy (AFM) indentation was performed using a Bruker Multimode 9 AFM with a Nanoscope V controller. An initial image was acquired using tapping mode. The indentation and imaging experiments were performed in ambient conditions.

Effect of Applied Force on Indentation Depth

50-Poly(S-*r*-DCPD)

A range of applied forces between 0.3 μN to 3.8 μN were examined through indentation of the surface of samples of 50-poly(S-*r*-DCPD). A total of 6 replicates were obtained for each applied force. A force curve was generated for each indentation to extract maximum applied force. Once the sample was modified, a high-resolution AFM image was acquired of the area, to extract the depth using Nanoscope Analysis and the projected area using Gwyddion. The data acquired are tabulated below and shown in Figure 2B in the main text. For the graphical representation the mean indentation depths are presented, and the error bars are represented by the standard error of the mean. An applied force of 3.8 μN generated an indentation that exceeded 25 nm in depth, which is greater than the depths of indentations used recorded for use in data storage.

Supplementary Table S4: Table of applied force, projected area, and indentation depth for 50-poly(S-*r*-DCPD). Standard Error (SERR) is recorded as 1 standard deviation from the mean divided by square root of number of samples.

Applied Force (μN)	Projected Area (nm^2)	Indentation Depth (nm)	SERR (nm)
0.27	284	0.7	0.5
0.57	449	1.8	0.1
1.1	815	4.9	1.2
1.8	1341	10.1	0.8
2.3	1951	14.8	0.4
2.8	2090	20.1	1.0
3.3	3350	23.4	0.9
3.8	4464	28.2	0.8

50-Poly(S-*r*-CPD)

A range of applied forces between 0.3 μN to 5.2 μN were examined on the surface of samples of 50-poly(S-*r*-CPD). A total of 6 replicates were obtained for each applied force. A force curve was generated for each indentation to extract maximum applied force. Once the sample was modified, a high-resolution AFM image was acquired of the area to extract the depth using Nanoscope Analysis and the projected area using Gwyddion. The data acquired are tabulated below and shown in Figure 2C in the main text. For the graphical representation, the mean indentation depths are presented, and the error bars are represented by the standard error of the mean. An applied force of 4.6 μN and 5.2 μN generated an indentation that exceeded 25 nm in depth, which is greater than the depths of indentations used recorded for use in data storage. For 50-poly(S-*r*-CPD) a force of 0.3 μN resulted in no measurable indentation.

Supplementary Table S5: Table of applied force, projected area, and indentation depth for 50-poly(S-*r*-CPD). Standard Error (SERR) is recorded as 1 standard deviation from the mean divided by square root of number of samples.

Applied Force (μN)	Projected Area (nm^2)	Indentation Depth (nm)	SERR (nm)
0.33	-	-	0
0.66	501	1.8	0.1
1.3	846	5.0	0.2
2.1	1427	7.9	0.1
2.8	1919	12.2	0.1
3.4	2496	16.5	0.4
3.9	3395	22.3	0.5
4.6	4199	27.8	1.7

SEM and EDX analysis of AFM tips before and after indentation process

SEM images were acquired of AFM tips (MikroMasch HQ:NSC15) used during the initial nanoindentation studies to assess wear, contamination, or damage of the tips due to use. No major wear or damaged was observed. EDX spectroscopy was used at four sites on the tip, with a point much higher up (control) to assess contamination due to contact with the polymer. Only silicon was detected by EDX and no sulfur from polymer contamination was observed.

Supplementary Figure S3: SEM images of AFM tips post experiments compared with an unused AFM tip. A) after reading, writing and erasing across 14 samples making approximately 800 indentations B) after reading, writing across 4 samples making approximately 400 indentations C) Unused AFM tip.

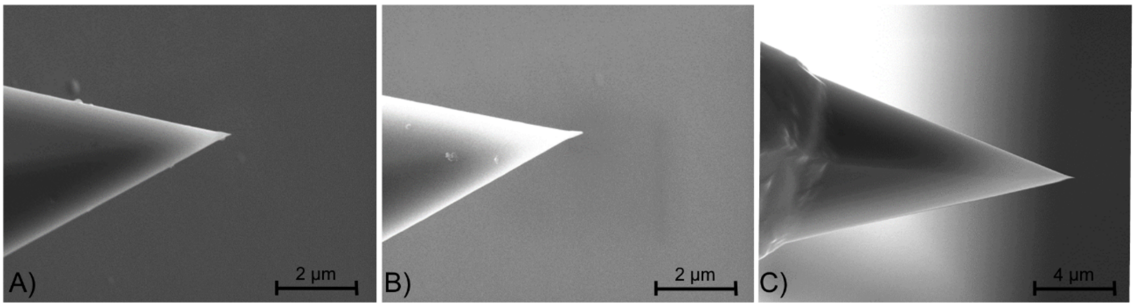

SEM images of AFM tip after polymer modification, with sites of EDX analysis highlighted.

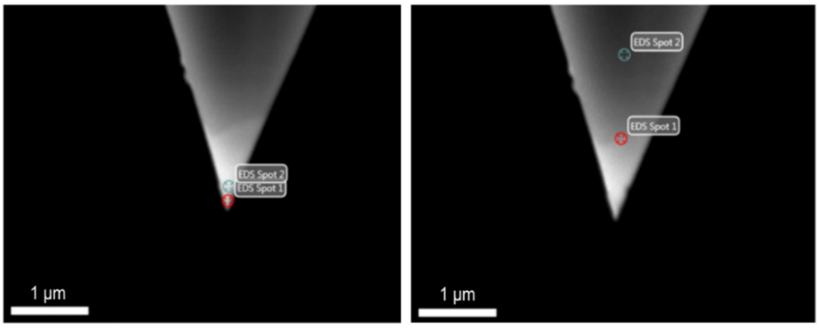

Representative EDX Spectra for all points analyzed. The sulfur K_α peak is usually at 2.307 keV, so evidence of polymer contamination was observed.

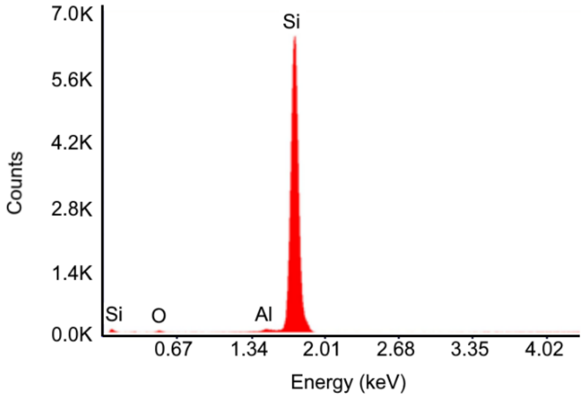

Improving data density using an AFM tip with a higher aspect ratio

To improve density of data and increase the bits (indentations) per square inch, a tip with a higher aspect ratio was trialed (Bruker VTESPA 300). Indentations were undertaken following the general methods. Indentations were applied to both 50-poly(S-*r*-DCPD) and 50-poly(S-*r*-CPD). Forces were measured to be approximately 200 nN. The probe was calibrated for their Spring Constant (K) and the deflection sensitivity using previously mentioned methods.

VTESPA-300	
F_0 (kHz)	325.674
K_{Sader} (N/m)	39.0
Deflection Sensitivity (nm/V)	46.7

Supplementary Figure S4: SEM micrograph of the VTESPA-300 AFM tip apex:

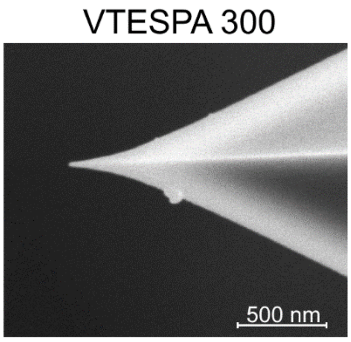

Supplementary Figure S5: AFM images and cross-sectional profile of indentations:

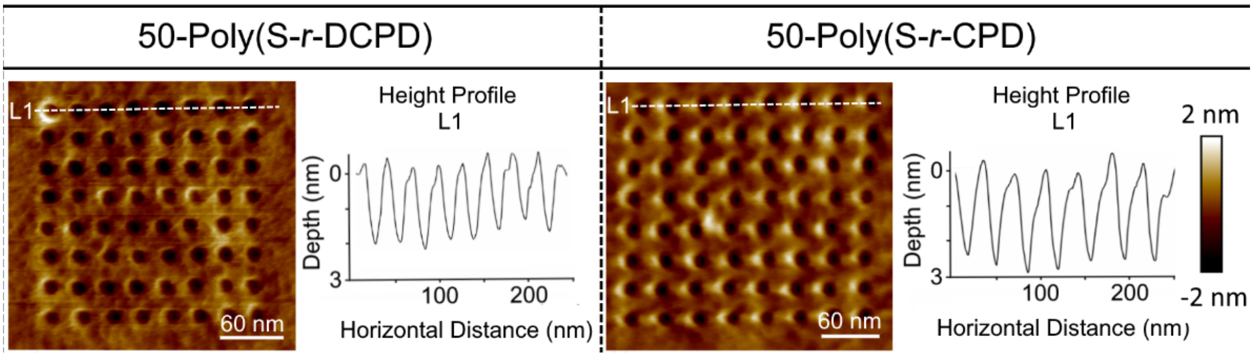

With higher control over spacing and indentation size using the Bruker VTESPA 300 tip, the unoptimized data density was calculated to be 0.9 Tb/in^2. With and optimized pitch of 22 nm the maximum data density using this tip and conditions were calculated to be ~ 1.5 Tb/in^2.

Encoding information on 50-poly(S-*r*-DCPD) using the VTESPA-300 tip

Using the VTESPA-300 tip, indentations were applied to the surface of 50-Poly(S-r-DCPD) in either a binary or a ternary coding language. For binary, the standard ASCII format was followed, with a start bit added in for a clear indication of where each 8-bit strand starts. For the ternary encoding method, a 0,1,2 format was used, as shown in the table below. The ternary coding reduced the area required for each character by using 3 cells with 3 potential states for a character, increasing data density 4 fold.

Ternary code (27 character set) using 3 numbers for each letter of the English alphabet. In this coding, 0 = no indentation, 1 = indentation of 0.3-1.0 nm depth, 2 = indentation of 1.5-2.5 nm depth.

A	B	C	D	E	F	G	H	I	J	K	L	M	N	O	P	Q	R	S	T	U	V	W	X	Y	Z
0	1	2	0	2	0	0	1	1	2	2	0	0	1	1	2	2	0	1	1	2	2	1	2	1	1
0	1	2	1	1	0	0	1	1	2	2	1	2	0	2	0	1	2	2	0	0	1	0	0	2	0
0	1	2	2	0	1	2	0	2	0	1	0	0	1	1	2	2	1	0	2	1	1	0	0	2	0

Supplementary Figure S6: Selection of Figure 3 in the main text illustrating data storage using binary and ternary coding:

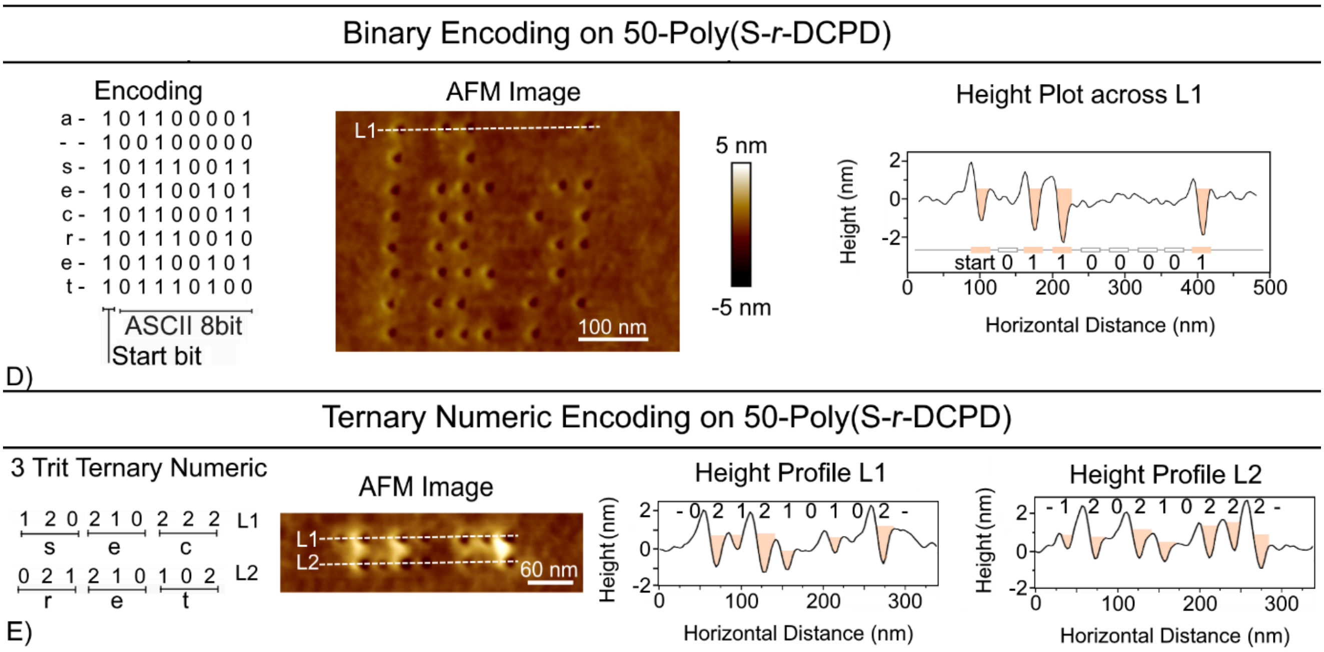

Supplementary Table S6. Calculated data density for above figures:

**Method**	**Min Area of Character (nm^2)**	**Area of Character (in^2)**	**Density (Characters/ in^2)**
Ternary	1627	2.5×10^{-12}	4.0×10^{11}
Binary	7050	9.9×10^{-12}	1.0×10^{11}

Supplementary Table S7. Calculated theoretical maximum data densities for both Ternary and Binary methods of encoding data.

**Method**	**Min Area of Character (nm^2)**	**Area of Character (in^2)**	**Density (Characters/ in^2)**
Ternary	1401	2.2×10^{-12}	4.6×10^{11}
Binary	3696	5.8×10^{-12}	1.7×10^{11}

Additional examples of storing information on 50-poly(S-*r*-DCPD) using an alternative ternary code

Nanoindentation was carried out on 50-poly(S-*r*-DCPD) according to the general method using the Bruker VTESPA 300 tip. Forces of approximately 0.7 μN, 1.1 μN and 1.6 μN were used to install indentations of three distinct depths that could be resolved by AFM. The depths were used to encode for “1”, “2” or “3” in the ternary coding system indicated below.

Alternative ternary Code with 3 numbers assigned to each letter of the English alphabet

A	B	C	D	E	F	G	H	I	J	K	L	M	N	O	P	Q	R	S	T	U	V	W	X	Y	Z
1	2	3	1	3	1	1	2	2	3	3	1	1	2	2	3	3	1	2	2	3	3	2	3	2	2
1	2	3	2	2	1	1	2	2	3	3	2	3	1	3	1	2	3	3	1	1	2	1	1	3	1
1	2	3	3	1	2	3	1	3	1	2	1	1	2	2	3	3	2	1	3	2	2	1	1	3	1

Supplementary Figure S7. AFM image of calibration indents for encoding, and cross-sectional plots showing depth and shape of indentation.

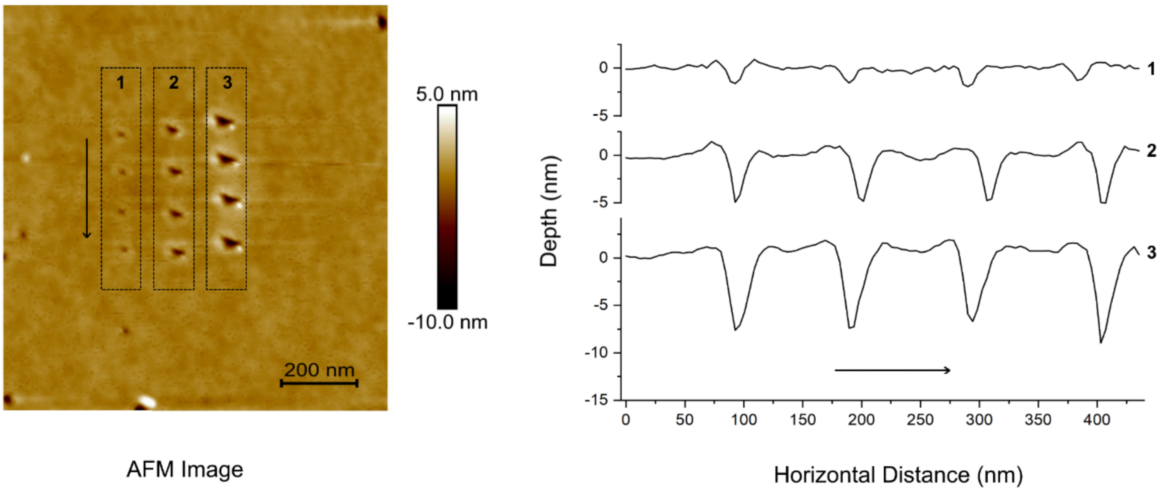

Average depths, standard error, and accepted range of indentation for a numerical representation.

Numerical Representation	Average Depth (nm)	Standard Error (nm)	Accepted Range (nm)
1	1.9	0.1	1.0-2.6
2	4.2	0.3	2.6- 5.8
3	7.2	0.6	5.8-10.8

Writing a word on 50-poly(S-*r*-DCPD) using an alternative ternary code

Using the previously defined key for encoding and the calibration grid shown on the previous page, the word HELLO was encoded onto the surface of 50-poly(S-*r*-DCPD).
AFM image of encoded word, and cross -section plots showing depth and shape of indentation.

Supplementary Figure S8

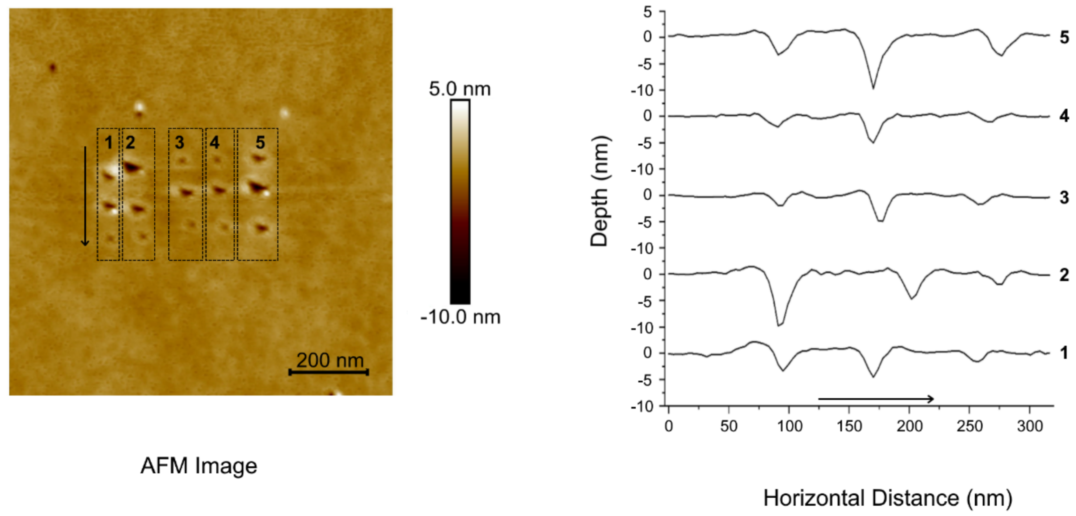

Average depths, of indentation per line and corresponding numerical pattern representation.

	1	2	3	4	5
**Depth (nm)**	3.0	8.5	1.1	2.1	3.8
	4.8	5.7	4.7	4.8	10.4
	2.0	2.4	1.2	1.3	3.1
**Numeric Pattern**	221	321	121	121	232
**Letters**	H	E	L	L	O

Stability of information encoded on 50-poly(S-*r*-DCPD)

The samples from page S17-S18 were stored in a plastic petri-dish under ambient conditions. They were imaged again using AFM after 1 week and 3 months. The AFM images indicate the code could be read and that no change was observed over this period.

Supplementary Figure S9. AFM images of encoded information for the initial process, after 1 week of storages and over 3 months of storage

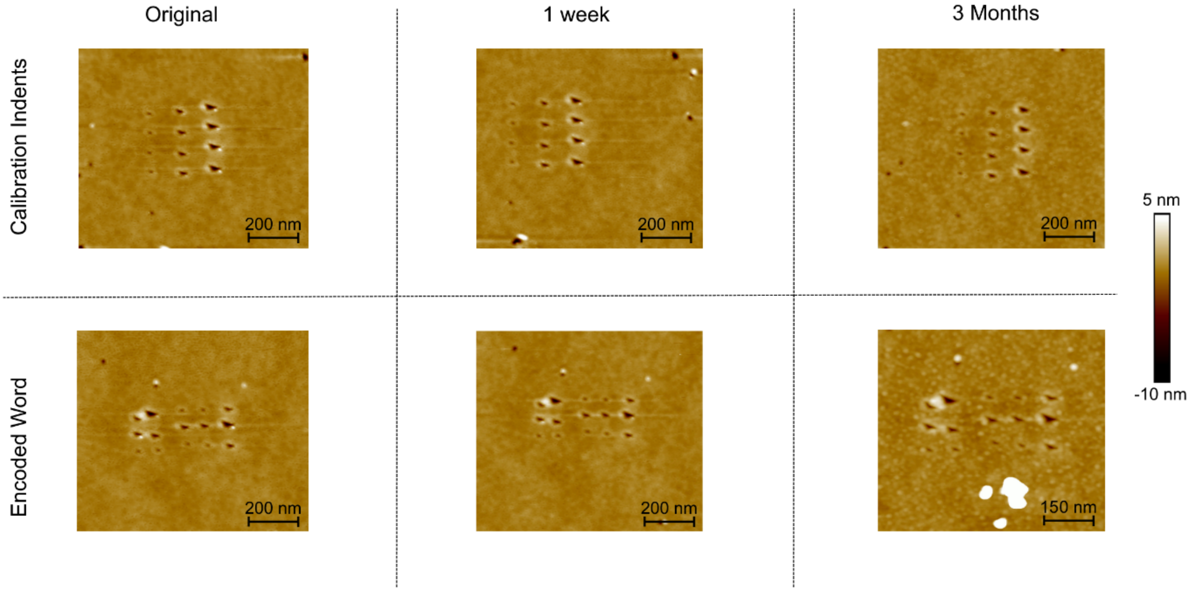

Reading encoded information after 1 week of storage:

Numerical Representation	Average Depth (nm)	Standard Error (nm)	Accepted Range (nm)
1	1.7	0.2	1.0-2.6
2	4.5	0.1	2.6- 5.8
3	8.0	0.3	5.8-10.8

	1	2	3	4	5
**Depth (nm)**	2.9	8.9	1.7	1.8	4.2
	4.7	5.1	4.2	3.8	8.3
	1.9	1.95	1.9	1.8	3.4
**Numeric Pattern**	221	321	121	121	232
**Letters**	H	E	L	L	O

Reading encoded information after 3 months of storage:

Numerical Representation	Average Depth (nm)	Standard Error (nm)	Accepted Range (nm)
1	2.0	0.1	1.0-2.6
2	4.2	0.2	2.6- 5.8
3	7.3	0.1	5.8-10.8

	1	2	3	4	5
**Depth (nm)**	3.9	8.5	1.9	1.9	3.9
	4.2	4.5	4.4	4	9.1
	1.9	2.5	2.1	2.1	4.5
**Numeric Pattern**	221	321	121	121	232
**Letters**	H	E	L	L	O

Erasing studies

Indentations were applied to the surface to the surface of 50-poly(S-*r*-DCPD) and 50-poly(S-*r*-CPD) following the general method. Grids of 16 indentations (18 nm to 27 nm) were constructed using the Mikromasch HQ:NSC tip. AFM images were acquired of the indentations to measure the depth of indentation before heating. The polymer sample with indentation pattern was then placed on a metal heating block on a hot plate and monitored with the FLIR thermal imaging camera. The temperature of the sample was recorded every 30 seconds for 2 minutes after reaching the desired temperature. After heating, the samples were allowed to return to room temperature and left for 24 hours before imaging using AFM to compare the depth of the remaining indentations after heating. The results are plotted and tabulated below.

Erasing indentations in 50-Poly(S-*r*-DCPD)

Supplementary Figure S10. Left: Average indentation depth (nm) before and after heating for a series of different temperatures. Right: change in indentation depth (nm) after heating, and % of indentation depth erased for a series of temperatures. The erasing process onset was observed to be 140 °C and above.

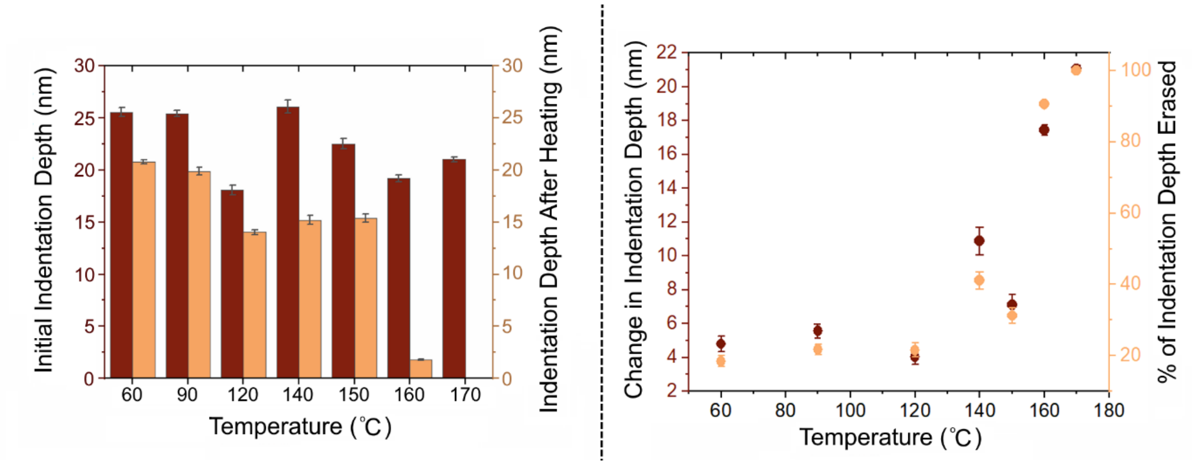

Summary of results for erasing indents in 50-poly(S-*r*-DCPD) (16 replicates at each temperature):

Temperature (°C)	Depth erased (nm)	Standard error (nm)	% of indentation depth erased	Standard error (%)
60 ± 5	4.8	0.5	18.4	1.6
90 ± 1	5.6	0.4	21.7	1.5
120 ± 1	4.0	0.4	21.6	2.0
140 ± 2	7.1	0.6	31.2	2.2
150 ± 2	10.9	0.8	41.1	2.4
160 ± 1	17.4	0.3	90.7	0.4
170 ± 1	21.0	0.2	100.0	0.0

Supplementary Figure S11. AFM images of indentation before and after heating 50-poly(S-*r*-DCPD) to temperatures between 60-170 °C. The heating was applied in each case for 2 minutes. A change in the indents is clear at 140 °C and higher. The indents are completely filled in (erased) at 170 °C.

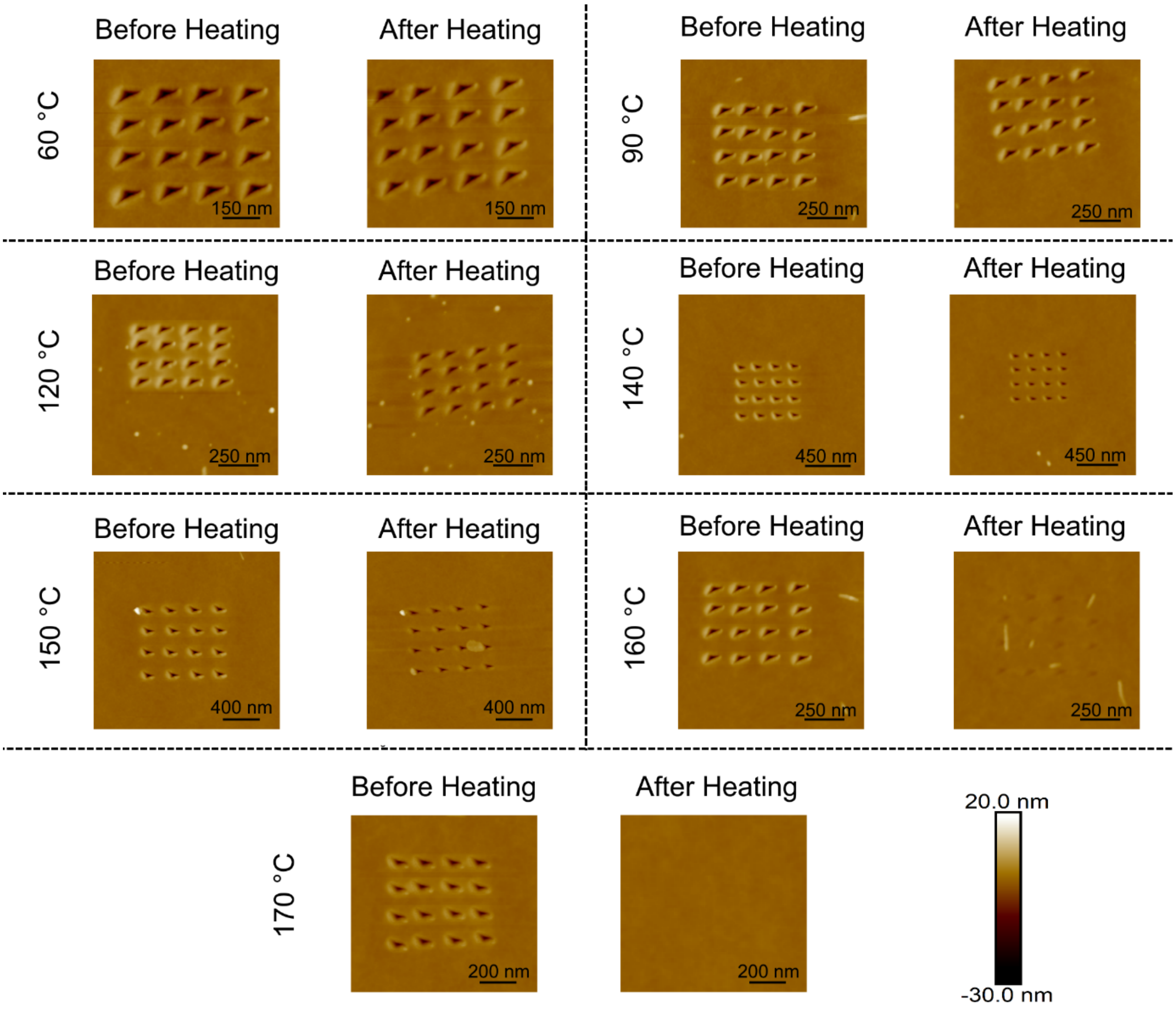

Erasing indentations in 50-poly(S-*r*-CPD)

Supplementary Figure S12. Left: Average indentation depth before and after heating for a series of different temperatures. Right: change in indentation depth (nm) after heating, and % of indentation depth erased for a series of temperatures. No change was observed at 60°C, but then the indentations were partially erased at higher temperatures.

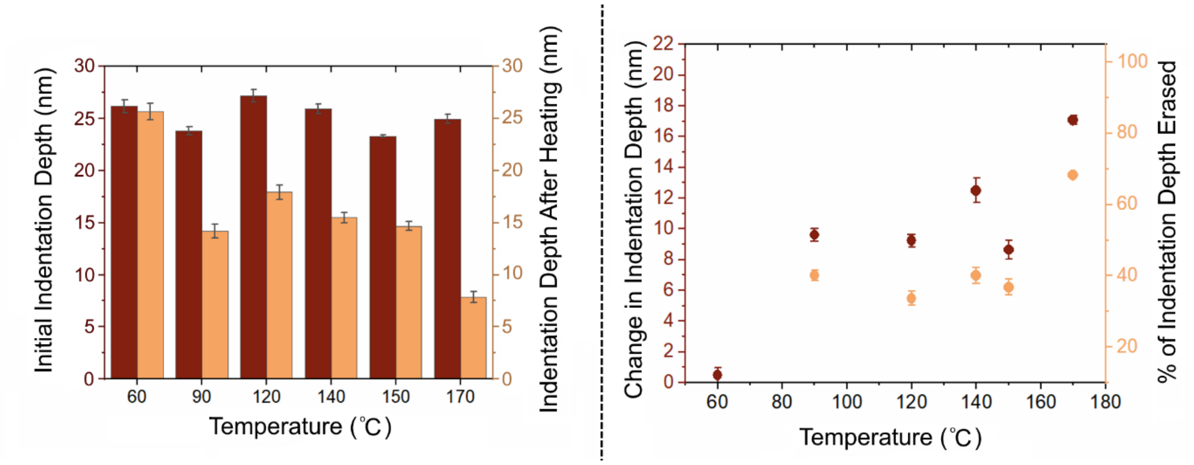

Summary of results for erasing indents in 50-poly(S-*r*-CPD) (16 replicates at each temperature):

**Temperature (°C)**	**Depth erased (nm)**	**Standard error (nm)**	**% of indentation depth erased**	**Standard error (%)**
60 ± 2	0.5	0.7	1.5	2.7
90 ± 1	9.6	0.8	40.1	3.1
120 ± 1	9.2	0.8	33.7	2.6
140 ± 1	12.5	0.6	40.1	2.2
150 ± 1	8.6	0.6	36.8	2.0
170 ± 1	17.1	0.7	68.3	2.2

Supplementary Figure S13. AFM images of indentations before and after heating the sample of the 50-poly(S-*r*-CPD) to temperatures between 60-170 °C. While the indentation depth was clearly reduced, incomplete erasing was observed for 50-poly(S-*r*-CPD), even at 170 °C.

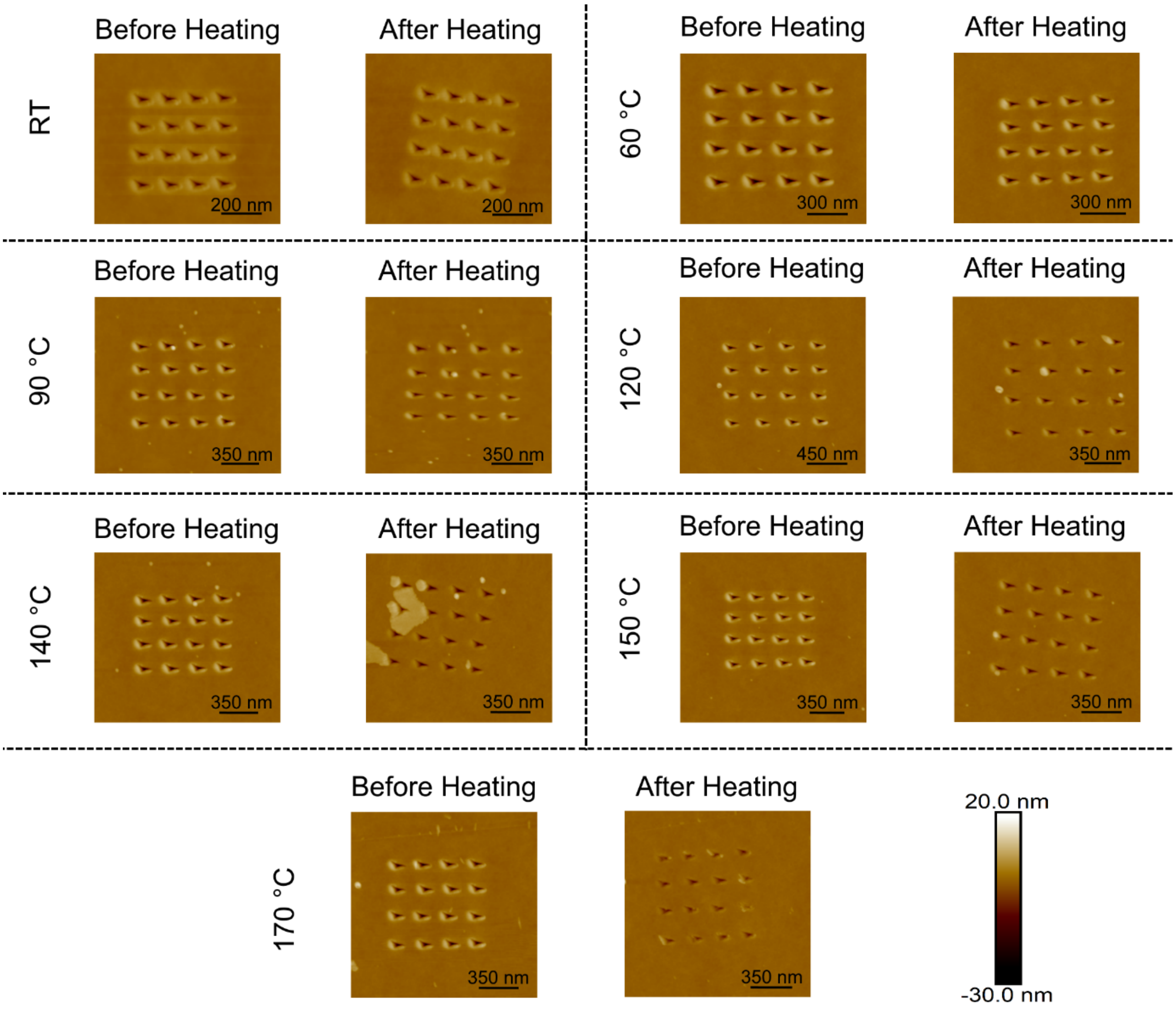

Re-writing data after erasing indentations on 50-poly(S-*r*-DCPD)

In the erasing studies, it was found that heating to 170 °C for two minutes could erase indentations with a depth of 17-18 nm. The approximate force used to generate those indentations was 4.2 μN. The next series of experiments were to assess the possibility of re-writing information on the polymer surface after erasing the indentations. After the erasing process, the surface was analyzed using AFM to find the same area, and then the indents were re-applied to the surface. The indentations could indeed be re-written onto the surface post heating to erase the initial information.

Supplementary Figure S14. Example AFM images showing the erasing and re-writing process in the same area of the polymer surface:

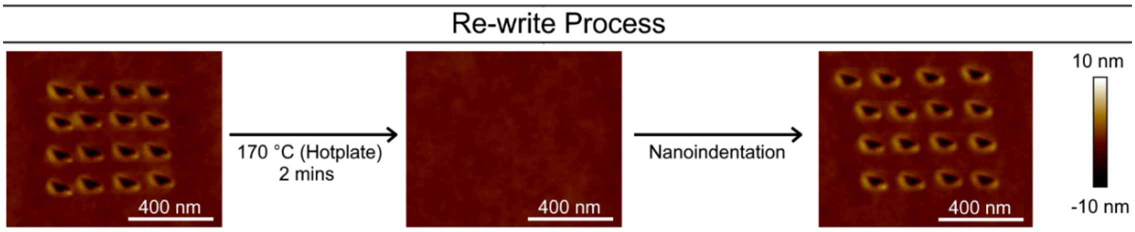

Supplementary Figure S15. Example AFM images of the erasing and re-writing process for 2 iterations. The cross-section plots are shown for the dashed lines. Tabulated depths for all indentations are shown on the following page.

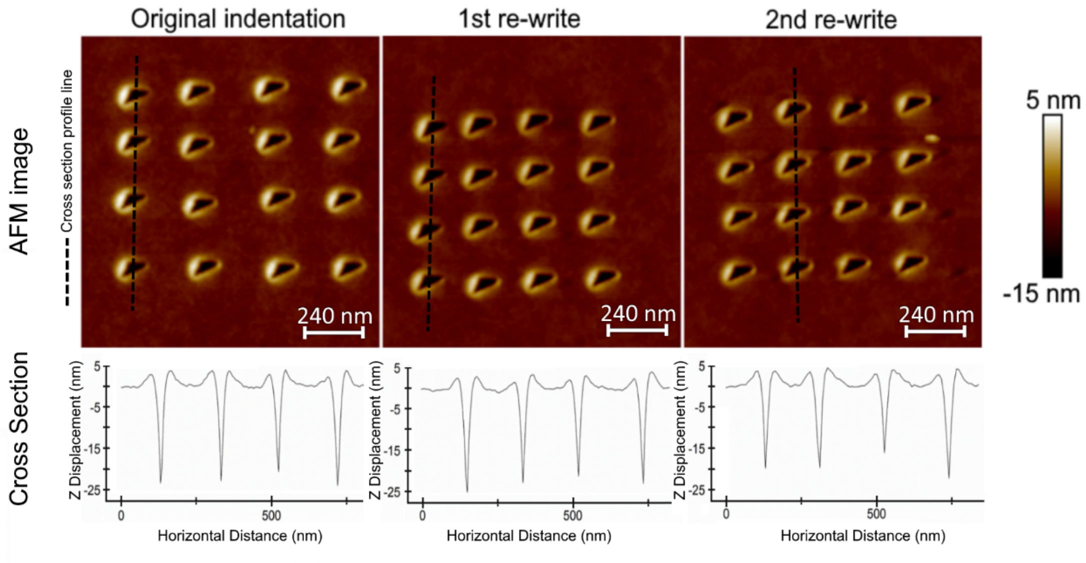

Supplementary Table S8. Tabulated depths of indentations during initial writing process, 1st re-write after heating and 2nd re-write after heating.

indent #	Initial Write Depth (nm)	1st Replication Depth (nm)	2nd Replication Depth (nm)
1	20.5	24.1	26.1
2	20.3	22.5	25.7
3	18.1	23.1	25.8
4	18.9	23.2	23.5
5	21.4	25.7	23.4
6	20.6	22.5	23.7
7	20.7	24.3	25.5
8	18.9	24.1	24.2
9	24.0	25.5	26.2
10	20.1	24.8	20.4
11	22.6	20.8	23.0
12	18.1	25.3	23.4
13	20.6	26.0	22.5
14	23.2	26.5	23.8
15	20.0	23.8	23.3
16	21.9	22.8	26.5
Average	20.6	24.1	24.2
SD	1.7	1.5	1.6
Error	0.4	0.4	0.4

Indentations on films of 50-poly(S-*r*-DCPD)

Films of 50-poly(S-*r*-DCPD) were prepared on a glass slide. The film was made to a thickness of 800 – 900 nm by using a heated glass pipette to transfer a drop of the viscous pre-polymer to a pre-cut, silicon wafer (8 mm × 8 mm). Then a glass slide was placed on top of the pre-polymer drop, which caused it to spread out into a film. A screw clamp was used to secure the assembly as shown in the diagram below. The clamped stack was then placed in an oven to cure for 12 h at 140 °C. After curing, the material was cooled to room temperature and the silicon wafer removed to reveal the polymer film, adhered to the glass slide. The film was thermally polished to reduce surface roughness by placing the glass slide on a hotplate (preheated to 150 °C) for 2 minutes. Notably the final polymer film is clear, in contrast to the cured bulk polymer, which is black.

Supplementary Figure S16.

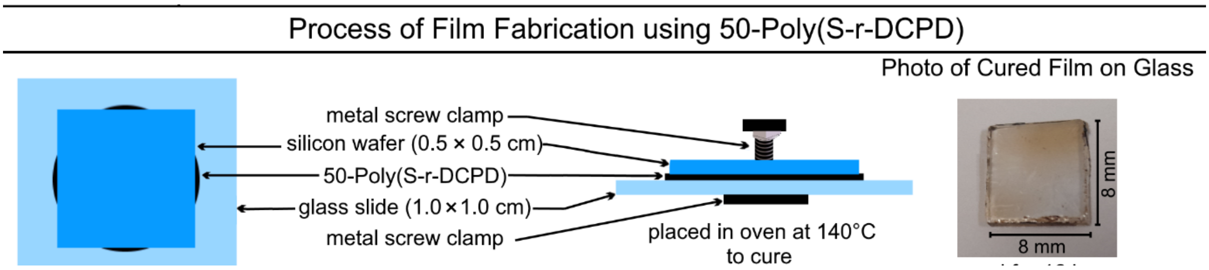

Film Thickness Measurements:

The thickness profile of the films was measured using stylus profilometry over 1.5 mm of the film. These measurements were taken over the area of modification. Thickness profiles were accumulated over a width of 100 µm and then averaged.

Surface Roughness Measurements:

An 8 µm × 8 µm area image was acquired of the polymer sample surfaces at the location of modification. Roughness parameters such as Average (R_a), and root mean square (RMS, R_q), were extracted through a roughness analysis performed on the AFM image using Nanoscope Analysis. The roughness was measured to be $R_a = 0.30$ nm and $R_q = 0.43$ nm.

Indentation:

Atomic force microscopy (AFM) indentation was performed using a Bruker Multimode 9 AFM with a Nanoscope V controller. An initial image was acquired using tapping mode, of the area of interest. The indentation and imaging experiments were performed in ambient conditions. Hardness was examined over a total 37 indentation, using a force of approximately 215 – 265 nN generating indentations with a depth of approximately 1-2 nm. After indentation had been achieved an image of the modified area was acquired by AFM. The indentations of the thin film were then compared to the bulk material, by undertaking hardness measurements as described using the following equation:[4]

$$H_{AFM} = \frac{F_{n,\ max}}{A_P} \qquad \text{Equation 3}$$

Where $F_{n,max}$ is the maximum applied force of the tip to the surface during indentation and A_p is the projected area calculated using Gwyddion.

50-poly(S-*r*-DCPD)	$F_{n,max}$ (μN)	Ave A_p (nm^2)	Ave H_{AFM} (MPa)
Polymer film	0.27	518	511
Bulk polymer	0.33	354	932

Erasing:

The sample of 50-poly(S-*r*-DCPD) with indentations was placed on a metal heating block on a hot plate. Temperatures were measured using a FLIR thermal imaging camera. After reaching the desired temperature, the sample was heated for 10 seconds. After heating, the samples were allowed to return to room temperature and left for 30 minutes before imaging using AFM to compare the depth of the remaining indentations after heating.

Supplementary Figure S17. Selection of Figure 5 from the main text showing the polymer film thickness and writing and erasing on the polymer film. Surface roughness and hardness data are provided. Indentations with a depth of 1 nm could be erased by heating for 10 seconds at 140 °C. The writing, reading, and erasing process was repeated 4 times.

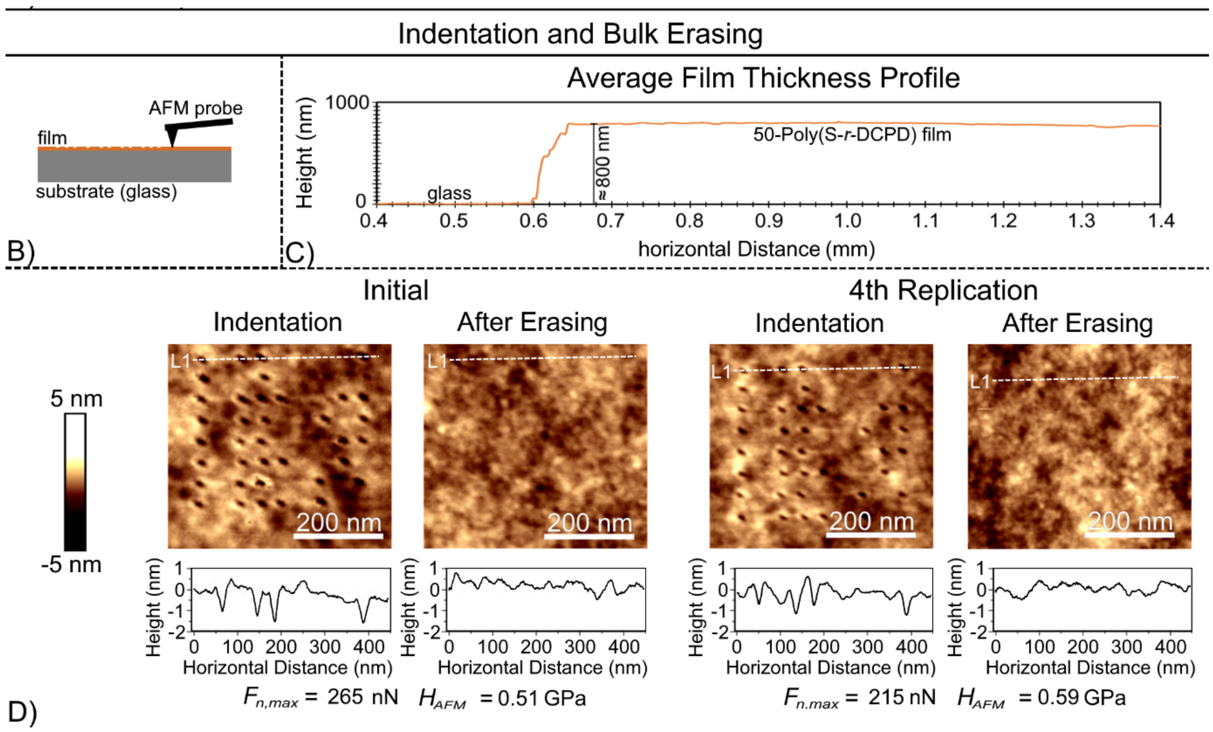

SEM images of indentations on 50-poly(S-*r*-DCPD) (bulk polymer)

Indentation was performed on samples of 50-poly(S-*r*-DCPD) using a Bruker Multimode 9 AFM with a Nanoscope V controller. The indentation and imaging experiments were performed in ambient conditions using tapping mode. Indentations were performed in arrays of 6 × 18, 6 × 18 and 10 × 25 indentations, using applied forces of approximately 1.2 µN to 3.7 µN (see imaging on following page). The indentation depths were approximately 3 nm for 1.2 µN force and16 nm for 3.7 µN force.

For encoding information in binary, the standard ASCII format was followed, with a start bit added in for a clear indication of where each 8-bit strand starts. An applied force of 1.2 µN was used to generate indentations of approximately 3 nm in depth. A standard Mikromasch HQ:NSC 15 probe was used for this experiment.

After indentation had been achieved an image of the modified area was acquired by AFM. An SEM image was also acquired to compare with the AFM image. The images are shown on the following page.

SEM reading rate

A maximum speed of 0.6 kb/s for the reading rate could be achieved using a dwell time of 10 µs per pixel for a 2048 × 1768-pixel resolution image with each encoded bit size equating to ~171 pixels. The image was captured using a magnification of 60000 ×, a working distance of 4.0 mm, and a voltage of 5.0 kV.

$$\text{Time taken to read 1 bit} = 171 \ \ pixels/bit \times 10\,\mu s/pixel$$

$$\text{Time taken to read 1 bit} = 1710\,\mu s \ or\ 0.00171\,s$$

$$Reading\ speed\left(\frac{bits}{s}\right) of\ reading\ (imaging) = \frac{1}{0.00171\,second/bit} = 0.6\,kb/s$$

Supplementary Figure S18. Left: AFM images of arrays of indentations on the surface of 50-poly(S-*r*-DCPD) with forces of indentation ranging from 1.2 µN to 3.7 µN giving a range of indentation depths (approx. 3 nm for 1.2 µN force and16 nm for 3.7 µN force). Right: the corresponding high resolution SEM images showcasing the ability to image and read the encoded information using an orthogonal readout method.

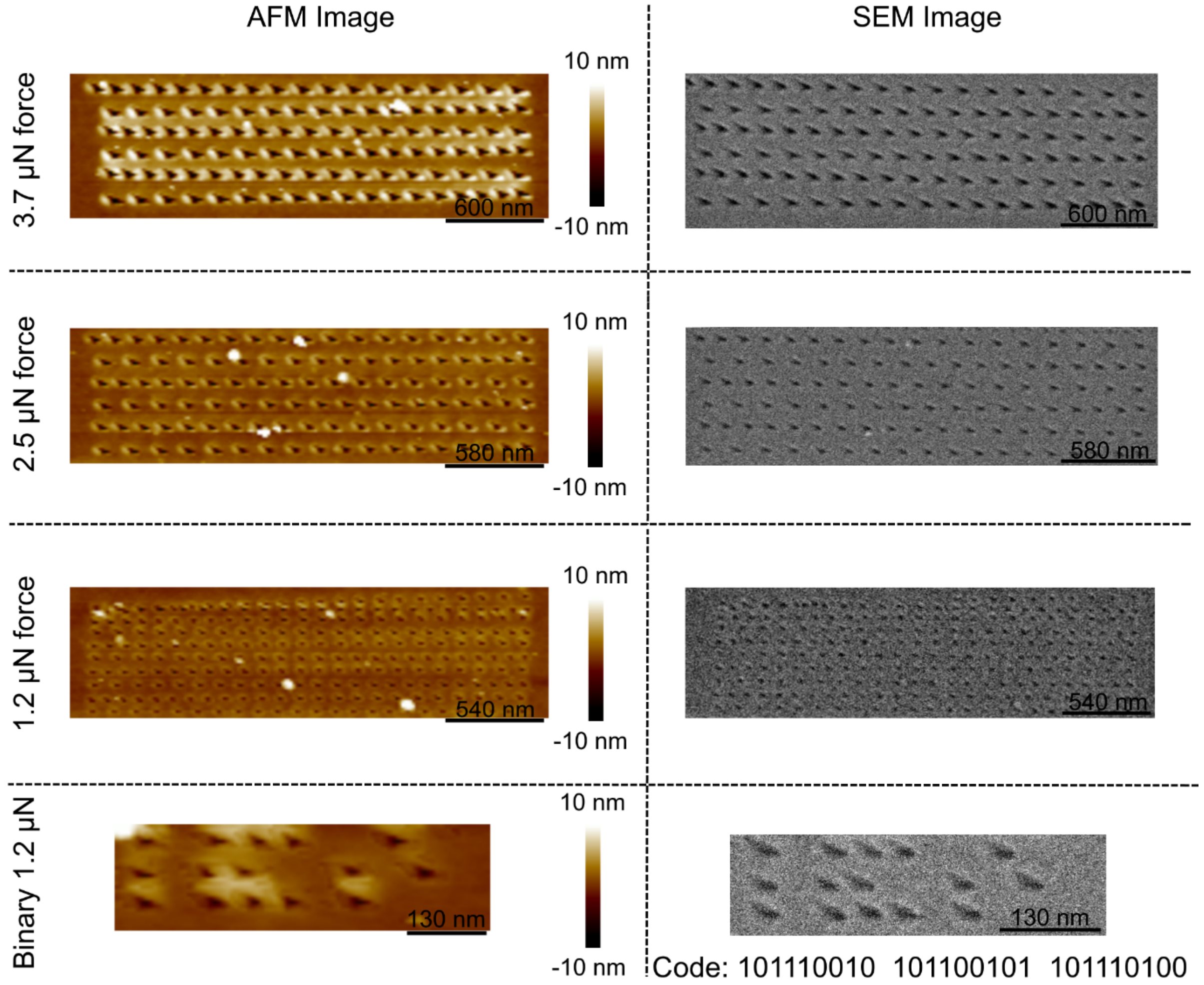

SEM images of indentations on 50-Poly(S-*r*-DCPD) polymer films

Films of 50-Poly(S-*r*-DCPD) were prepared following methods described on page S27. Indentation was performed using a Bruker Multimode 9 AFM with a Nanoscope V controller. The indentation and imaging experiments were performed in ambient conditions. Indentations were performed in an array of 10 × 10, and then as the force decreased the phrase “a secret” was encoded into the surface, using applied forces of approximately 2.2 μN and 275 nN giving a range of indentation depths (approx. 1.5 nm for 275 nN and 12 nm for 2.2 μN). For binary coding, the standard ASCII format was followed, with a start bit added in for a clear indication of where each 8-bit strand starts. A Bruker VTESPA 300 tip was used for this experiment. After indentation, the modified area was imaged using AFM. An SEM image with was also acquired to compare with the AFM image. A maximum speed of 0.6 kb/s for the reading rate could be achieved using a dwell time of 10 μs per pixel for a 2048 × 1768 pixel resolution image with each bit equating to ~171 pixels. The image was captured using a magnification of 60000 ×, a working distance of 4.0 mm, spot size of 2.0 and Voltage if 5.0 kV.

Supplementary Figure S19. A selection from Figure 5 in the main text is shown below to illustrate the complementary imaging of the encoded information using either AFM or SEM.

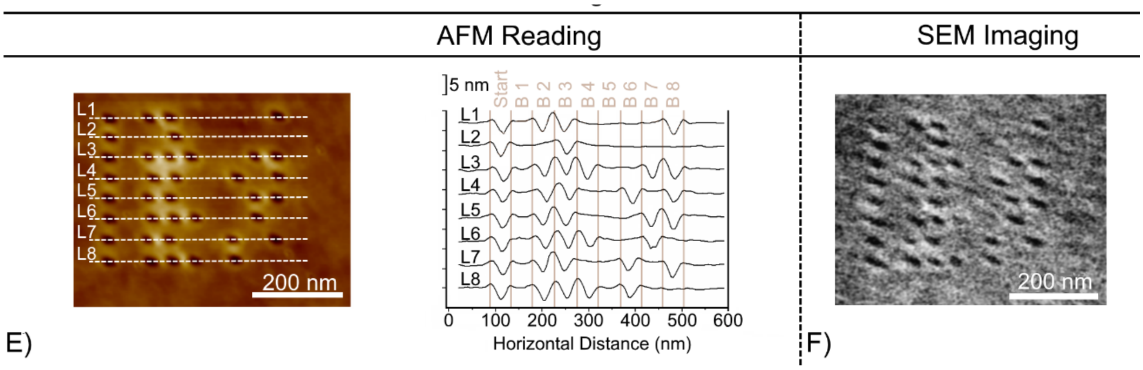

Stability of polymer films over repeated heating and cooling cycles

Preparation of Films

Films of 50-poly(S-*r*-DCPD) were prepared on a glass slide as outlined on page S27, films of thicknesses of ~550 nm and ~ 7.5 μm were studied.

Characterisation

Film thickness was determined using stylus profilometry over a 100 μm area. The two films were approximately 550 nm and 7.5 μm respectively.

Heating and cooling cycles

The stability of the polymer films were evaluated after repeated heating and cooling cycles. The film was heated to 140 °C on a hotplate for 10 seconds and then removed from the heat and cooled to room temperature. This cycle was repeated 30 times.

Analysis

After the heating a cooling cycles, AFM was used to assess surface deformation and roughness, and also to see if any particles (such as elemental sulfur) form after repeated heating and cooling. SEM and EDX spectroscopy were used to assess the elemental distribution across the polymer film, and Raman spectroscopy was used to assess if any elemental sulfur formed by degradation of the polymer.

Results

AFM analysis indicated there was no significant change in the surface roughness over the heating and cooling cycles. SEM and EDX indicated a relatively uniform elemental distribution before and after heating, with no evident change. Raman spectroscopy did not show any evidence for formation of elemental sulfur (which has 3 strong characteristic peaks between 200 and 500 cm^{-1})[3] The tabulated data is provided below and on the following page.

Supplementary Table S9. AFM roughness of the surface before and after 30 cycles of heating to 140 °C

		R_a	SD	R_q	SD
550 nm film	Before	0.25	0.03	0.39	0.10
	After	0.26	0.00	0.37	0.02
7.5 μm film	Before	0.30	0.09	0.55	0.20
	After	0.27	0.02	0.40	0.08

Supplementary Figure S20. Analysis of 550 nm film of 50-poly(S-*r*-DCPD) after 30 heating and cooling cycles:

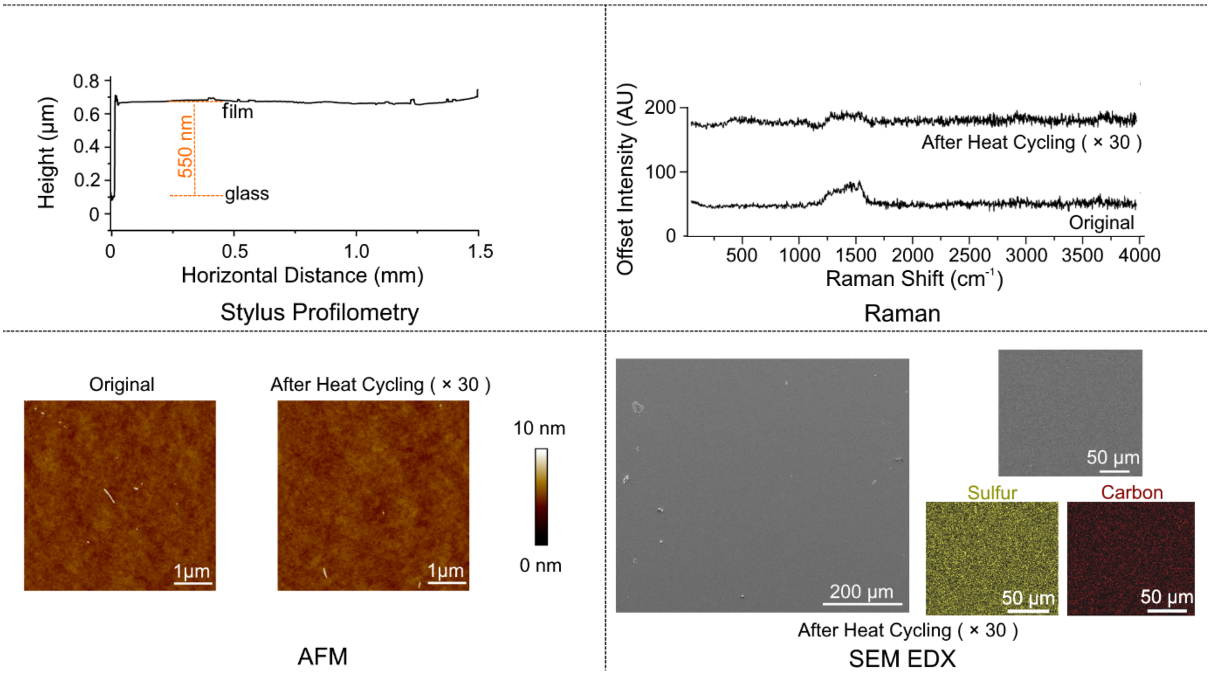

Supplementary Figure S21. Analysis of 7.5 μm film of 50-poly(S-*r*-DCPD) after 30 heating and cooling cycles:

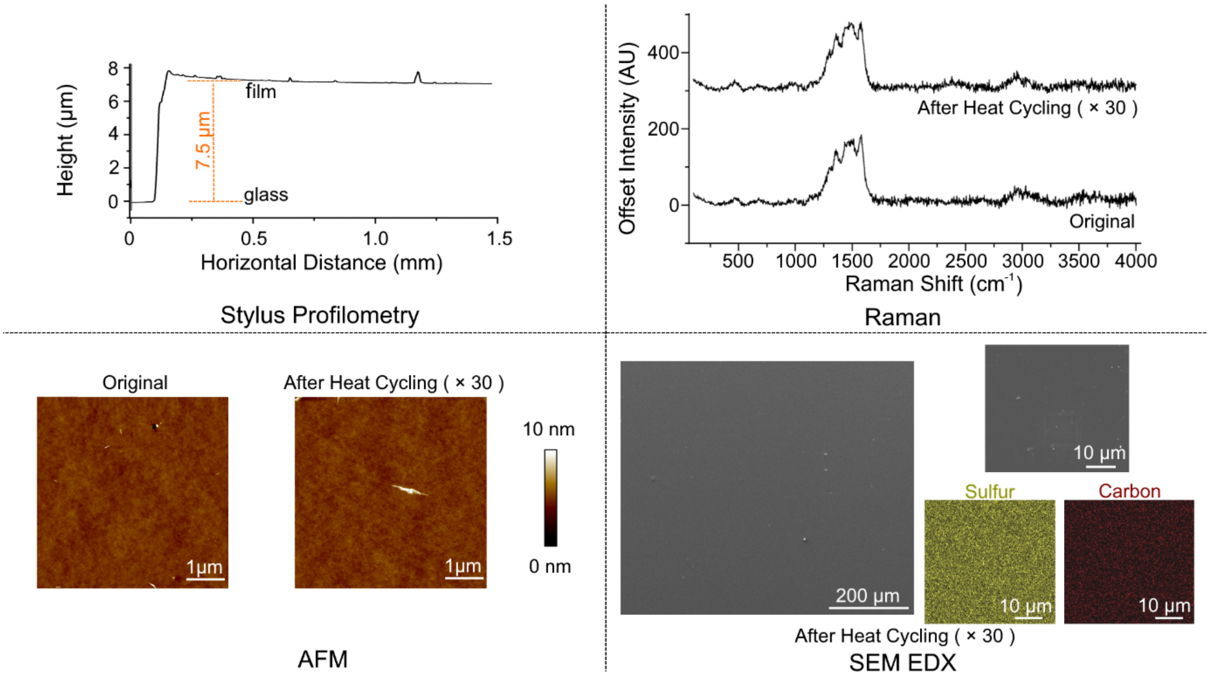

Encoding information on 50-poly(S-*r*-DCPD) films of different thicknesses

Polymer films with thicknesses of 550 nm and 7.5 μm were prepared as described on page S27 and S33.

Atomic force microscopy (AFM) indentation was performed using a Bruker Multimode 9 AFM with a Nanoscope V controller. Writing and reading the code using AFM on the 50-poly(S-*r*-DCPD) film was undertaken using a Bruker VTESPA 300 tip with a calibrated average spring constant of 34.6 N/m and average deflection sensitivity of 49.3 nm/V. An initial image was acquired using tapping mode. The indentation and imaging experiments were performed in ambient conditions. Hardness was examined over a total 37 indentation, using a force of approximately 193 nN and 143 nN generating indentations with a depth of approximately 2 nm. After indentation had been achieved an image of the modified area was acquired by AFM. Hardness was also measured for both films.

Supplementary Figure S22.

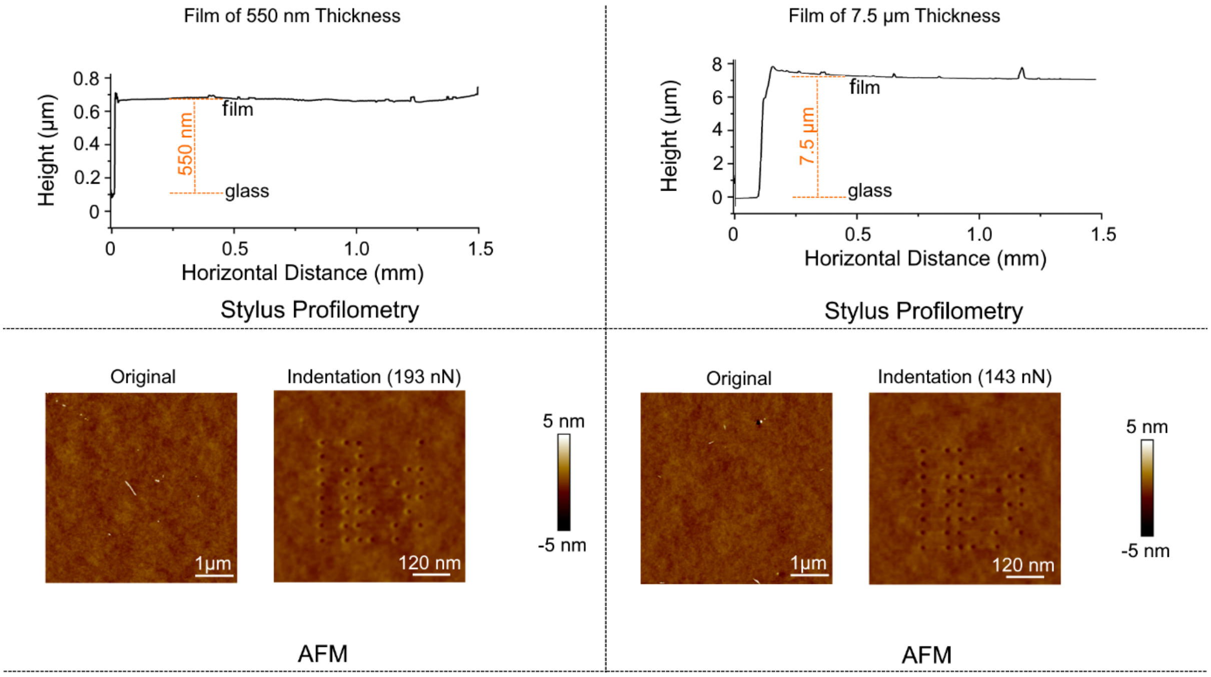

Calculated hardness of polymer films of 50-poly(S-*r*-DCPD)

**50-poly(S-*r*-DCPD)**	**$F_{n,max}$ (nN)**	**Ave A_p (nm^2)**	**Ave H_{AFM} (MPa)**
550 nm film	193	209	961
7.5 um film	143	195	769

Reproducibility in encoding information on 50-poly(S-*r*-DCPD) film

Encoding Information

Using the 550 nm thin film of 50-poly(S-*r*-DCPD) made in the previous section, atomic force microscopy (AFM) indentation was performed using a Bruker Multimode 9 AFM with a Nanoscope V controller. An initial image was acquired using tapping mode, of the area of interest. The indentation and imaging experiments were performed in ambient conditions. The phrase “a secret” was encoded using binary ASCII as described previously, using a force of approximately 193 nN generating indentations with a depth of approximately 2 nm. After indentation had been achieved, an image of the modified area was acquired by AFM. This process was replicated 4 times with in a 2 μm by 2 μm square area. The readout was consistent across all images, unambiguously revealing the same encoded information. This experiment demonstrates the reproducibility of the writing and reading process for mechanical data storage on 50-poly(S-*r*-DCPD) polymer films.

Supplementary Figure S23.

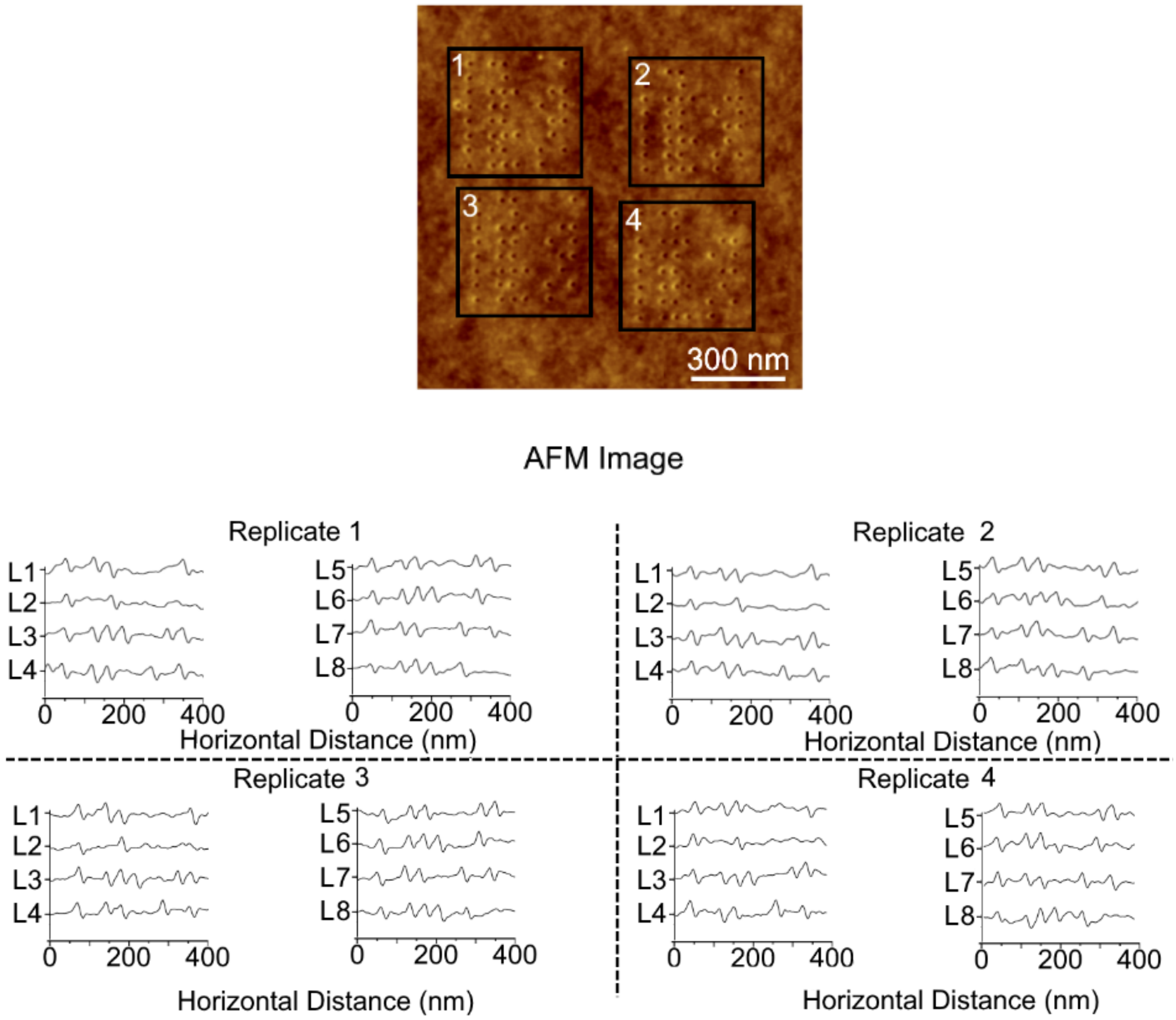

Polymer films using spin coating

Solutions of the 50-poly(S-*r*-DCPD) prepolymer (before curing) were prepared in chloroform at concentrations 50 mg/g and 100 mg/g. A volume of 100 µL was added drop wise on to the pre-cut substrates. Glass, a silicon wafer, and poly imide tape (all approximately 2 cm by 2 cm) were used as substrates to test the feasibility of spin coating on materials of different type. The stage was rotated at 2000 RPM over a 1-minute interval. Three and four repetitions of deposition were carried out to build up an appropriate thickness, as specified in the figure below. The samples where then cured in an oven set to 140°C for 24 hours. The resulting surface was imaged using a camera (left) and microscope at 10 x magnification (right). The cured polymer coating was uneven after curing, indicating spin coating is complicated by problems with beading and uneven wetting on glass, silicon, and poly imide. For this reason, 50-poly(S-*r*-DCPD) is best fashioned into films using the drop casting method described on page S27

Supplementary Figure S24.

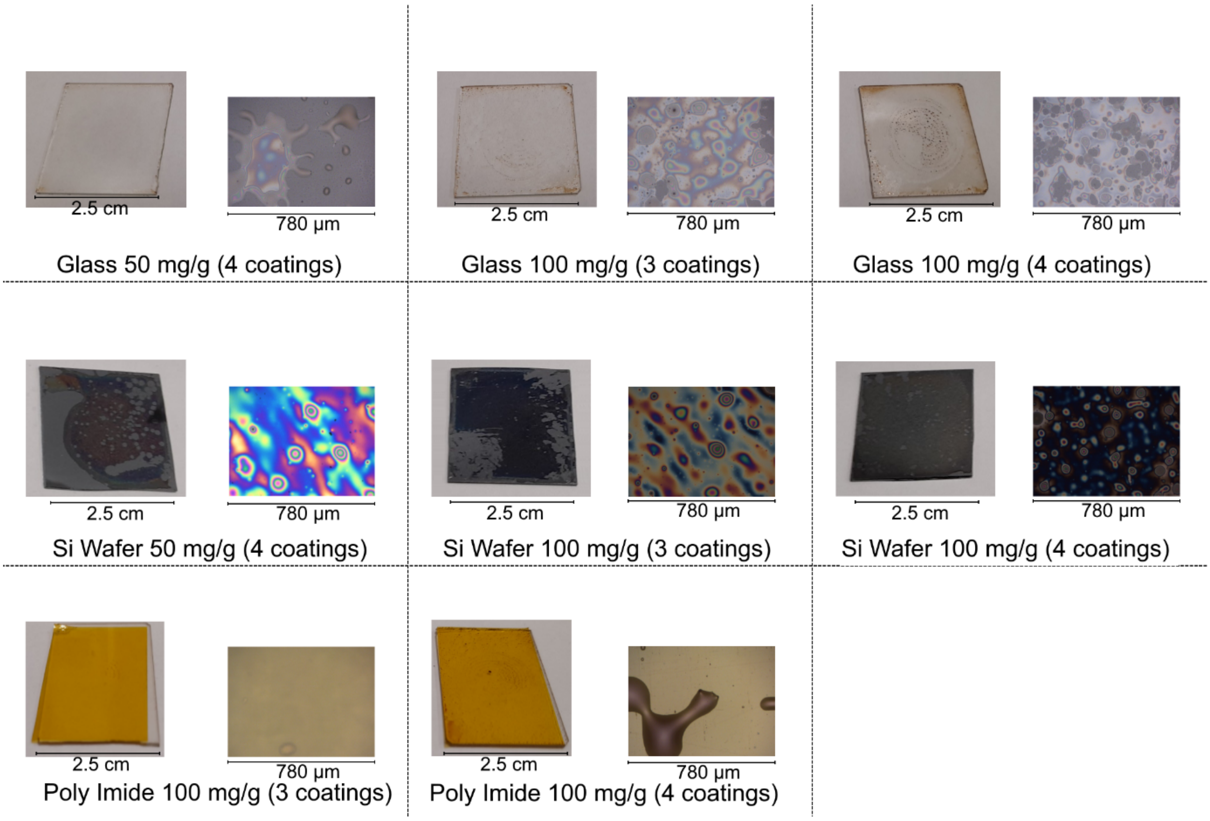

References

1. Sader, J. E.; Borgani, R.; Gibson, C. T.; Haviland, D. B.; Higgins, M. J.; Kilpatrick, J. I.; Lu, J.; Mulvaney, P.; Shearer, C. J.; Slattery, A. D.; Thorén, P.-A.; Tran, J.; Zhang, H.; Zhang, H.; Zheng, T., A virtual instrument to standardise the calibration of atomic force microscope cantilevers. *Review of Scientific Instruments* **2016,** *87*, 093711.
2. Mann, M.; Zhang, B.; Tonkin, S. J.; Gibson, C. T.; Jia, Z.; Hasell, T.; Chalker, J. M., Processes for coating surfaces with a copolymer made from sulfur and dicyclopentadiene. *Polymer Chemistry* **2022,** *13*, 1320-1327.
3. Tonkin, S. J.; Pham, L. N.; Gascooke, J. R.; Johnston, M. R.; Coote, M. L.; Gibson, C. T.; Chalker, J. M., Thermal Imaging and Clandestine Surveillance using Low-Cost Polymers with Long-Wave Infrared Transparency. *Adv. Optical Mater.* **2023,** *11*, 2300058.
4. Caron, A., Quantitative Hardness Measurement by Instrumented AFM-indentation. *J. Vis. Exp.* **2016,** *117*, 54706.
5. Rosato, D. V.; Rosato, D. V. *Plastics Engineered Product Design.* Elsevier, New York, 2003.
6. Kontomaris, S. V.; Malamou, A., Hertz model or Oliver & Pharr analysis? Tutorial regarding AFM nanoindentation experiments on biological samples. *Mater. Res. Express* **2020,** *7*, 033001.
